# Serum Fatty Acid Profiles Across Normoglycemia, Prediabetes, and Type 2 Diabetes: Associations with Glycemic and Cardiometabolic Parameters in a Kazakh Population

**DOI:** 10.3390/ijms27219702

**Published:** 2026-10-29

**Authors:** Maxat Toishimanov, Aksholpan Shokan, Ivan Voitsekhovskiy, Bakyt Kanapiyanov, Nurgul Myrzabayeva, Alma Nurtazina

**Affiliations:** 1 Kazakhstan-Japan Innovative Center, Kazakh National Agrarian Research University, Almaty 050010, Kazakhstan; 507957@kaznaru.edu.kz (M.T.);; 2 Institute of Genetic and Physiology, Almaty 050040, Kazakhstan; 3 Faculty of Biology and Biotechnology, Al-Farabi Kazakh National University, Almaty 050038, Kazakhstan; 4 Department of Epidemiology and Biostatistics, Semey Medical University, Semey 071400, Kazakhstan; 5 Department of Propaedeutics of Internal Diseases, Semey Medical University, Semey 071400, Kazakhstan

**Keywords:** serum fatty acids, fatty acid profile, prediabetes, type 2 diabetes mellitus, HbA1c, GC–MS, compositional data, Kazakhstan

## Abstract

Population-specific data on serum fatty acids across the progression to type 2 diabetes mellitus (T2DM) are limited. In this exploratory cross-sectional study, serum fatty acids were determined by gas chromatography–mass spectrometry in 177 adults from Kazakhstan (45 normoglycemic, 114 with prediabetes, 18 with T2DM) and expressed as percentages of the quantified profile of the 13 components detected in ≥50% of samples. No fatty acid differed between glycemic groups after false discovery rate correction. In controls and participants with prediabetes, oleic and palmitoleic acids correlated with serum triglycerides (ρ = +0.65 and +0.32; q ≤ 0.005), and both associations persisted after adjustment for age, sex and adiposity and after exclusion of implausible profiles arising from a peak-assignment inconsistency. Across all participants, linoleic acid correlated inversely with glycated hemoglobin (HbA1c; ρ = −0.21, q = 0.033) in unadjusted analyses only. An association of heptadecanoic acid with HbA1c depended on its non-detection in part of the samples and was neither established nor excluded. Covariates were not recorded for the T2DM group, HbA1c was recorded with limited precision for most participants, and adjusted analyses were restricted to 107 participants without diabetes. The study describes serum fatty acid composition in this population and generates hypotheses.

## 1. Introduction

Type 2 diabetes mellitus (T2DM) is among the fastest-growing chronic disease burdens worldwide. According to the eleventh edition of the IDF Diabetes Atlas, an estimated 589 million adults aged 20–79 years (11.1% of the adult population) were living with diabetes in 2024, a figure projected to rise to 853 million by 2050; approximately 635 million adults are estimated to have impaired glucose tolerance, and some 252 million cases of diabetes remain undiagnosed [1]. An independent analysis of Global Burden of Disease data estimated that approximately 462 million people were affected by T2DM in 2017, corresponding to 6.28% of the world’s population, with prevalence forecast to continue rising [2]. The burden of undetected disease falls disproportionately on low- and middle-income countries: in a pooled analysis of 117 population-based studies, the proportion of diabetes that was previously undiagnosed and detected only through survey screening ranged from 30% in the high-income Western region to 66% in south Asia, and reliance on fasting glucose alone was shown to underestimate prevalence in these settings [3].

Kazakhstan exemplifies this pattern. Analysis of the Unified National Electronic Health System, covering 475,539 registered patients, showed that the prevalence of type 1 and type 2 diabetes increased 1.7-fold between 2014 and 2019 [4], and national registry data for 2018–2021 documented a rise in recorded prevalence from 2142.2 to 3743.9 per 100,000 population, accompanied by a more than 1.5-fold increase in diabetes-related mortality [5]. Population-based measurement consistently exceeds registry-based estimates: in a WHO STEPwise survey of 4753 adults from four geographically distinct regions, the survey-weighted prevalence of T2DM was 8.0% and that of impaired fasting glucose 1.9%, with 54% of diabetes cases newly detected at the time of the survey and reduced high-density lipoprotein (HDL) cholesterol emerging as an independent predictor of impaired fasting glucose (OR 3.72, 95% CI 1.68–8.23) [6]; comparable underdiagnosis had been reported earlier in the Astana region [7]. A substantial share of the metabolic burden in this population therefore lies upstream of clinical diagnosis, in individuals whose routine laboratory results are either normal or only marginally abnormal.

Prediabetes is the stage at which this trajectory is still modifiable. Classical estimates place annual conversion from prediabetes to diabetes at 5–10%, with a similar proportion reverting to normoglycemia [8], and an individual-participant pooled analysis of 19 prospective cohorts (76,092 participants, median follow-up 9.8 years) found that reversion to normoglycemia (36.1%) was in fact more frequent than progression to T2DM (12.5%) over ten years [9]. This plasticity does not make the state benign: meta-analyses covering more than one hundred cohorts show that prediabetes is associated with increased risk of cardiovascular disease and all-cause mortality [10,11], and an umbrella review of meta-analyses confirms elevated risks of microvascular and macrovascular complications before the diabetic threshold is crossed [12]. Identifying the metabolic reorganization that accompanies this interval therefore has both mechanistic and preventive value.

The markers used in routine practice are poorly suited to that task. Glycated hemoglobin (HbA1c) and fasting plasma glucose identify overlapping but non-identical populations; in a study of 3523 adults, agreement between the two criteria was only moderate (weighted κ = 0.55), and a large proportion of individuals classified as prediabetic by one criterion were normoglycemic by the other [13]. The conventional lipid panel is similarly insensitive at this stage, because the dyslipidemia of insulin resistance (modestly elevated triglycerides, reduced HDL cholesterol and a shift toward small dense low-density lipoprotein (LDL) particles) may coexist with total and LDL cholesterol concentrations that fall within the reference range [14]. Dietary assessment adds little: a dose–response meta-analysis of 13 cohorts comprising 361,686 participants and 11,865 incident cases found no association between reported intake of total saturated fat, palmitic acid (C16:0) or stearic acid (C18:0) and T2DM (total saturated fatty acids, HR 0.99, 95% CI 0.91–1.09) [15], even though circulating saturated fatty acid biomarkers are repeatedly associated with incident disease. Composition, rather than intake or aggregate lipid concentration, appears to carry the information.

Circulating fatty acids offer a direct read-out of that composition. Their proportions integrate dietary supply with endogenous de novo lipogenesis, chain elongation, desaturation and β-oxidation, and they are measured objectively rather than reported, avoiding the recall and portion-estimation error inherent in dietary instruments [16]. Individual species differ in their interpretation: odd-chain fatty acids such as pentadecanoic (C15:0) and heptadecanoic (C17:0) acids are not products of the standard even-chain synthetic pathway and show intake correlations up to 0.47 and 0.40, respectively, with intra-individual reproducibility coefficients of 0.50–0.70 [16], whereas polyunsaturated species including linoleic acid (C18:2n-6) track dietary intake more directly [17]. Ratios between substrate and product pairs within the profile have been used as proxies for elongase and desaturase activity and relate to insulin resistance and to the metabolically unhealthy phenotype: higher estimated stearoyl-CoA desaturase and δ6-desaturase activity, higher palmitic acid and lower linoleic acid characterize adverse metabolic status [18,19].

These associations, however, are not automatically transferable between populations. In the Multi-Ethnic Study of Atherosclerosis, plasma phospholipid fatty acid composition differed systematically between ethnic groups [20], and further analysis showed that acculturation and ethnic background jointly shape circulating n-6 and n-3 proportions independently of measured intake [21]. Reference data and effect estimates derived from European or North American cohorts may therefore misrepresent populations with different genetic backgrounds and dietary structures. To our knowledge, no serum fatty acid profile has been reported for an ethnic Kazakh or Central Asian population stratified by glycemic status, and the relationship of such a profile to glycemic and cardiometabolic parameters in this setting is unknown.

The present study was designed to address this gap. In a cross-sectional sample of adults from Kazakhstan classified as normoglycemic, with prediabetes or with T2DM, we determined serum fatty acids by gas chromatography–mass spectrometry and examined, first, whether fatty acid composition differs across glycemic categories; second, how individual fatty acids relate to glycated hemoglobin, fasting glucose, body mass index and the routine lipid panel; and third, whether any of these associations persist after adjustment for age, sex and adiposity and after control of the false discovery rate across all analytes. The analysis was exploratory. Our aim was to establish which components of the serum fatty acid profile, if any, distinguish intermediate hyperglycemia from normoglycemia in this under-studied population, and to define the metabolic domains they represent.

## 2. Results

### 2.1. Participant Characteristics and Available Clinical Data

The dataset contained 181 records of participants with archived serum. Two records were exact repeats of the records of two control participants, identical in every field, and each was retained once. Two samples from the prediabetes group in which no panel component was detected were excluded because sample preparation had failed. The analytical sample therefore comprised 177 participants: 45 normoglycemic controls, 114 participants with prediabetes and 18 participants with T2DM (Supplementary Figure S1).

Fasting plasma glucose (FPG) and glycated hemoglobin (HbA1c) were available for all 177 participants. A complete clinical profile (age, sex, anthropometry, blood pressure, serum lipids and creatinine) was recorded for 109 participants (24 controls and 85 with prediabetes), and anthropometric and blood-pressure measurements for 107 of them (24 and 83). The remaining 68 participants (21 controls, 29 with prediabetes and all 18 with T2DM) had FPG and HbA1c only. No participant with T2DM had any clinical variable other than FPG and HbA1c. Comparison across all three glycemic groups is therefore possible only for these two measures, and every analysis involving another clinical variable is restricted to controls and participants with prediabetes (Table 1; Supplementary Table S5).

The two glycemic measures were not of uniform quality. HbA1c was recorded to the nearest whole percentage point for 117 of the 177 participants, including 105 of the 109 with a complete clinical profile, and to two decimal places for the remainder. Among the 107 participants with anthropometric data, HbA1c was 5.0% or 6.0% in 102. For most of the sample, HbA1c is therefore in effect an ordinal measure of glycemic status rather than a continuous one. FPG was systematically lower in participants without a complete clinical profile than in those with one (median 3.85 versus 4.96 mmol/L in controls and 3.99 versus 5.28 mmol/L in prediabetes), which indicates that the two sets of glucose measurements are not directly comparable.

FPG and HbA1c differed across the three groups (*p* = 3.4 × 10^−9^ and 3.9 × 10^−31^), with all pairwise comparisons significant; because both measures enter the definition of the groups (Section 4.2), these differences follow from the classification. Among controls and participants with prediabetes, those with prediabetes had a larger waist circumference (Hodges–Lehmann shift +8.0 cm, 95% CI 3.0 to 14.0; *p* = 0.0031), higher body mass index (BMI; +2.25 kg/m^2^, 0.30 to 4.46; *p* = 0.025), higher total cholesterol (+0.52 mmol/L, 0.15 to 0.87; *p* = 0.011) and LDL cholesterol (+0.39 mmol/L, 0.06 to 0.74; *p* = 0.021), and were older (+5.0 years, 1.0 to 9.0; *p* = 0.028). Sex distribution, blood pressure, HDL cholesterol, triglycerides and creatinine did not differ significantly (Table 1).

### 2.2. Serum Fatty Acid Composition

**Figure 1:**
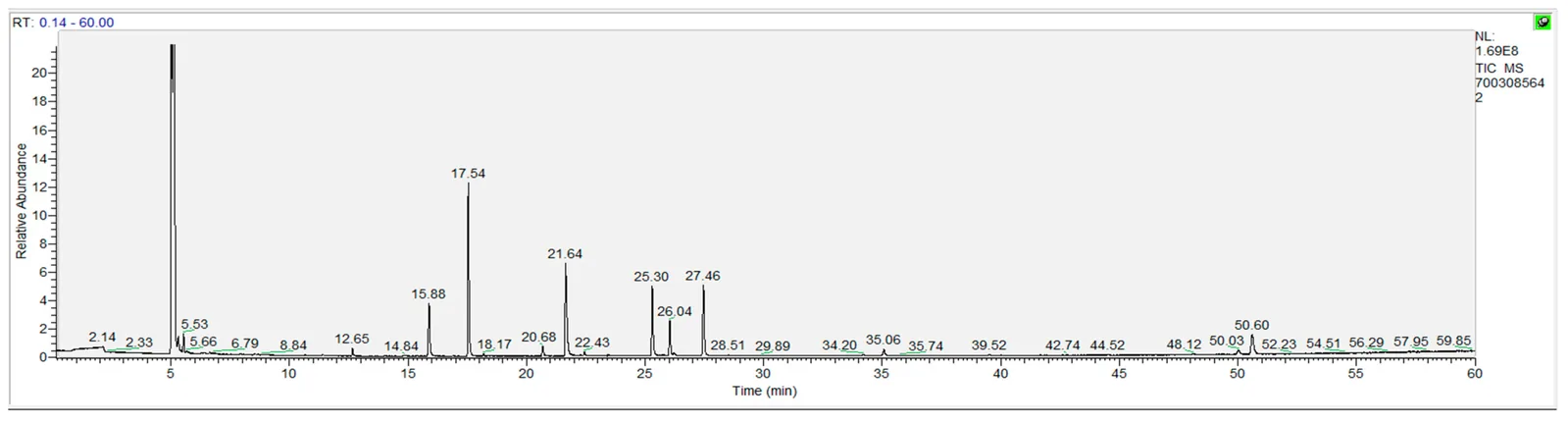
Representative total ion chromatogram of FAMEs obtained from a human serum sample by GC–MS.

Thirteen of the 37 panel components were detected (signal-to-noise ratio of at least 3 within the identification window) in at least half of the samples and were included in the compositional calculation and in the statistical analysis. All values are percentages of the quantified profile, that is, of the summed amount of these 13 components in each sample (Section 4.5). The 13 components accounted for a median of 99.9% (interquartile range 99.4–100.0%; minimum 97.0%) of the summed values of all detected panel components, so the restriction removes species rather than mass (Figure 1). The 24 excluded components include arachidonic acid (detected in 23% of samples), docosahexaenoic acid (7%), eicosapentaenoic acid (1%) and pentadecanoic acid (C15:0; 48%); their detection frequencies are given in Supplementary Table S2, and no inference is made about them.

Verification of the data revealed a sample-dependent inconsistency of peak assignment in the C18 region (Section 4.5). In 71 samples a single peak had been assigned to both C18:1 components, and in these samples stearic acid (C18:0) was implausibly high relative to palmitic acid; in 70 of the remaining 95 samples with both C18:1 components reported, stearic acid was not recorded at all. Stearic acid values are therefore not interpretable and are reported for completeness only. Applying three biochemical plausibility criteria defined in Section 4.5, 115 of the 177 profiles (30 controls, 74 with prediabetes and 11 with T2DM) were classified as plausible. Table 2 gives the composition in the full sample (A) and in these plausible profiles (B). Because stearic acid was not detected in most profiles even in the plausible subset, the composition of the C18 region remains incomplete, and Table 2 should not be read as a reference profile for this population.

In the full sample, five components differed nominally between the glycemic groups: linoleic acid (*p* = 0.019), heptadecanoic acid (C17:0; *p* = 0.022), caprylic acid (C8:0; *p* = 0.025), lauric acid (C12:0; *p* = 0.031) and palmitic acid (*p* = 0.039). None remained significant after Benjamini–Hochberg correction across the 13 components (smallest q = 0.100). Dunn post hoc testing (Table 3) localised the nominal differences in linoleic acid, C17:0 and palmitic acid to the comparison between controls and the T2DM group (Bonferroni-adjusted *p* = 0.023, 0.020 and 0.041), and those in C8:0 and C12:0 to the comparison between controls and prediabetes (*p* = 0.044 and 0.048). In the plausible profiles, only linoleic acid differed nominally (*p* = 0.0097; q = 0.126), between controls and prediabetes (*p* = 0.019) and between controls and T2DM (*p* = 0.049). Comparisons involving the T2DM group are descriptive: differences between controls and that group cannot be separated from differences in age, adiposity or treatment, none of which could be assessed in it.

### 2.3. Unadjusted Associations with Glycemic and Cardiometabolic Parameters

Unadjusted Spearman correlations are given in Table 4. Two correlations with HbA1c survived correction for multiple testing within that outcome (*n* = 177): C17:0 (ρ = −0.218, q = 0.033) and linoleic acid (ρ = −0.210, q = 0.033). The significance of the linoleic acid correlation depended on the correction of the C18:1 artefact, before which it was q = 0.071 (Section 2.6). No correlation with FPG survived correction.

Because lipid and anthropometric variables were not recorded for the T2DM group, the correlations with them concern controls and participants with prediabetes only. For triglycerides (*n* = 109), the strongest correlation was with oleic acid (C18:1n-9; ρ = +0.647, q < 0.001), followed by palmitoleic acid (C16:1; ρ = +0.319, q = 0.005) and, inversely, butyric acid (C4:0; ρ = −0.287, q = 0.011). Oleic acid was also correlated with BMI (ρ = +0.291, q = 0.030; *n* = 107), and linoleic acid with LDL cholesterol (ρ = +0.335, q = 0.005; *n* = 109). Stearic acid correlated inversely with triglycerides and LDL cholesterol but is not interpreted. No correlation with HDL cholesterol survived correction. The inverse correlations of minor saturated components with triglycerides accompany the higher oleic acid proportion and are consistent with the constant-sum constraint of compositional data.

In the 115 biochemically plausible profiles (Supplementary Table S7), the correlations of oleic and palmitoleic acids with triglycerides were stronger (ρ = +0.742, q < 0.001, and ρ = +0.332, q = 0.014; *n* = 72), and that of linoleic acid with HbA1c was similar (ρ = −0.272, q = 0.042; *n* = 115). The correlations of C17:0 with HbA1c (ρ = −0.161, q = 0.416) and of linoleic acid with LDL cholesterol (ρ = +0.283, q = 0.206) were not significant. Several further correlations reached q-values just below 0.05 in the plausible profiles only, for example, linoleic acid with FPG (ρ = −0.268, q = 0.050) and oleic, stearic and linoleic acids with HDL cholesterol (q = 0.049); because they were not observed in the full sample, they are reported in the Supplementary Materials but not interpreted.

### 2.4. Partial Correlations Adjusted for Age, Sex and Adiposity

Partial Spearman correlations adjusted for age, sex, BMI and waist circumference were computed for the 107 participants with all covariates (24 controls and 83 with prediabetes); the full matrix is given in Supplementary Table S3, and the associations that survived correction in either the full covariate subset or the plausible profiles are shown in Table 5. These analyses contain no participant with T2DM, and their results cannot be extended to that group.

The associations with triglycerides persisted after adjustment: oleic acid (r = +0.612, q < 0.001) and palmitoleic acid (r = +0.247, q = 0.027), both stronger in the 71 plausible profiles (r = +0.735, q < 0.001, and r = +0.290, q = 0.037). Linoleic acid remained correlated with LDL cholesterol (r = +0.389, q < 0.001; plausible profiles r = +0.347, q = 0.039). Palmitic acid and C4:0 correlated inversely with triglycerides. In the plausible profiles, linoleic acid also correlated inversely with triglycerides (r = −0.313, q = 0.026), whereas in the full covariate subset it did not (r = +0.082). No association with HbA1c or FPG survived correction after adjustment; the partial correlations of C17:0 and linoleic acid with HbA1c were r = −0.100 and r = +0.029.

### 2.5. Covariate-Adjusted Regression

In linear regression adjusted for age, sex and BMI (Table 6; *n* = 107), no fatty acid was associated with HbA1c or FPG after correction for multiple testing (smallest q = 0.344 for HbA1c and 0.813 for FPG). The standardized coefficient for C17:0 and HbA1c was β = −0.139 (95% CI −0.329 to +0.052; *p* = 0.152), and for linoleic acid β = +0.017 (−0.174 to +0.208; *p* = 0.859). Adding smoking status and antihypertensive medication did not change the estimates materially (C17:0, β = −0.130, *p* = 0.191). Because HbA1c took the value 5.0% or 6.0% in 102 of the 107 participants, these linear models are in effect comparisons of controls with participants with prediabetes rather than models of a continuous outcome. Like the partial correlations, they contain no participant with T2DM.

Multinomial logistic regression adjusted for age, sex and BMI, with the normoglycemic group as reference, could not be estimated: no covariate was recorded for any participant with T2DM, so the adjusted comparison of T2DM with controls contains no T2DM cases and the model is not identifiable. No adjusted odds ratio for prediabetes or T2DM is therefore reported.

### 2.6. Sensitivity Analyses

All sensitivity analyses are summarized in Supplementary Table S6.

Sensitivity analyses were performed to evaluate the influence of chromatographic peak-assignment inconsistencies, clinical data heterogeneity, detection-frequency thresholds, and compositional transformations on the observed associations (Supplementary Tables S4 and S6). Following correction of the duplicated C18:1 component, the absolute correlation coefficients increased in 48 of 78 fatty acid–outcome comparisons, decreased in 23, and remained unchanged in 7, with three associations changing their false discovery rate significance status. The association between oleic acid (C18:1n-9 cis) and triglycerides was the most consistent finding, remaining significant across all examined variants of the biochemical plausibility criteria (ρ = +0.58 to +0.80). Palmitoleic acid (C16:1) was also positively associated with triglycerides across these variants (ρ = +0.23 to +0.45), although statistical significance was retained in eight of eleven analyses. The inverse association between linoleic acid (C18:2n-6) and HbA1c was directionally consistent across the examined subsets (ρ = −0.21 to −0.39), but its statistical significance varied with sample selection and compositional transformation. In particular, the association was observed when linoleic acid was expressed relative to palmitic acid (ρ = −0.267, *p* = 0.0004), whereas it was attenuated or absent following centred log-ratio transformation (ρ = +0.102 and +0.003). Varying the detection-frequency threshold from 25% to 70% did not materially alter the principal correlation estimates, although this stability did not resolve the limitations associated with non-detected components. The inverse association between heptadecanoic acid (C17:0) and HbA1c was not significant in analyses restricted to biochemically plausible profiles (ρ = −0.13 to −0.20; q ≥ 0.20) or when only samples with detected C17:0 were considered (ρ = −0.020; *n* = 129). Similarly, censored regression showed an association in the full sample (β = −0.437, *p* = 0.008), but not in unaffected samples (β = −0.028, *p* = 0.84) or biochemically plausible profiles (β = −0.209, *p* = 0.18). Analyses accounting for differences in clinical data availability further demonstrated that associations with HbA1c were sensitive to the precision and distribution of glycemic measurements.

## 3. Discussion

### 3.1. Hierarchy of the Present Evidence

Because the number of analyses performed in this study exceeds the number of findings that withstood correction for multiple testing, and because verification of the data revealed an analytical problem that bears on every result, we set out the evidence explicitly before interpreting it (Table 7). The findings concern the 13 components detected in at least half of the samples, not the complete serum fatty acid profile, and each is labelled by the population and analysis from which it comes.

The best-supported observations concern triglycerides. Oleic and palmitoleic acids were correlated with serum triglycerides in unadjusted analyses, after adjustment for age, sex and adiposity, and in the biochemically plausible profiles, and the association of oleic acid was significant in every sensitivity analysis. These associations were observed in controls and participants with prediabetes, because lipids were not recorded for the T2DM group. The second level of support is the inverse association of linoleic acid with HbA1c. It was observed in unadjusted analyses across all three glycemic groups and was of similar size in every subset of the data, but it reached significance only after the C18:1 correction, was borderline in several subsets, was apparent as a lower ratio of linoleic to palmitic acid but not under centred log-ratio transformation, and was not observed in adjusted models. It has therefore not been shown to be independent of age, sex and adiposity.

Three further associations do not withstand verification. The association of linoleic acid with LDL cholesterol was inconsistent between samples affected and unaffected by the peak-assignment inconsistency, and that of oleic acid with BMI was not significant in the plausible profiles. The association of C17:0 with HbA1c, which the previous version of this manuscript presented as the central finding, depended on the non-detection of C17:0 in part of the samples and is neither established nor excluded by these data (Section 3.4). No fatty acid differed between glycemic groups after correction for multiple testing. We therefore do not claim that a fatty acid signature distinguishes normoglycemia, prediabetes and T2DM, and the data do not provide evidence of a fatty acid profile independently associated with diabetes status. The nominal group differences, and in particular those between controls and the T2DM group, are descriptive and cannot be separated from differences in age, adiposity or treatment.

### 3.2. Monounsaturated Fatty Acids and Triglycerides

The correlations of oleic and palmitoleic acids with triglycerides were the most consistent associations in this study. Oleic acid is the predominant fatty acid of triglycerides and of the triglyceride-rich lipoproteins, whereas linoleic acid predominates in cholesteryl esters and phospholipids. In an analysis of total serum fatty acids, a higher triglyceride concentration therefore shifts the composition toward oleic acid and away from other components, and part of the association is expected to reflect the mixture of lipid fractions rather than a change within any one of them. The inverse correlations of palmitic acid and several minor saturated components with triglycerides, and of linoleic acid in the plausible profiles, are consistent with this compositional interpretation. The association is informative about lipid handling and triglyceride-rich lipoproteins rather than about glycemia.

Palmitoleic acid is formed from palmitic acid by stearoyl-CoA desaturase and is a recognised marker of hepatic de novo lipogenesis, which is increased in insulin resistance. A serum fatty acid pattern combining higher palmitoleic acid and estimated stearoyl-CoA desaturase activity with lower linoleic acid has been reported to predict incident metabolic syndrome [22], and estimated stearoyl-CoA desaturase activity has been associated with incident diabetes [23]. The association of palmitoleic acid with triglycerides was the only one supported under every log-ratio approach. We did not compute desaturase indices, because the index usually used for the C18 pathway requires reliable quantification of stearic acid, which was not available here, and the association reported should be read as a compositional observation and not as a measure of enzyme activity.

### 3.3. Linoleic Acid

The inverse association of linoleic acid with HbA1c is directionally consistent with pooled evidence from 20 prospective cohorts comprising 39,740 adults, in which higher linoleic acid biomarkers were associated with a lower risk of T2DM (risk ratio per interquintile range 0.65, 95% CI 0.60–0.72) [24], and with the association of higher linoleic acid biomarkers with lower cardiovascular disease and mortality [25]. The present data support this association only in a limited form. Higher HbA1c was associated with a lower ratio of linoleic to palmitic acid in unadjusted analyses, consistently across subsets of the data and in the participants whose HbA1c was recorded as a continuous value, but the association was not apparent under centred log-ratio transformation or in the adjusted model, in which HbA1c was effectively binary. Because linoleic acid is essential and tracks dietary intake, a lower proportion at higher HbA1c may reflect differences in diet, relative dilution by endogenously synthesized fatty acids, or treatment in the T2DM group; the present data cannot distinguish between these possibilities, and the comparison with prospective cohorts indicates consistency of direction only.

A positive association of linoleic acid with LDL cholesterol is expected, since linoleic acid is the dominant fatty acid of cholesteryl esters and the analyte here is the total serum fatty acid composition rather than a single lipid fraction. In the present data, however, this association was inconsistent between samples affected and unaffected by the peak-assignment inconsistency and is not presented as a finding.

### 3.4. Heptadecanoic Acid and the Limits of Interpretation

Verification of the data showed that the inverse correlation of C17:0 with HbA1c in the full sample is carried by the frequency with which C17:0 was detected, which fell from 89% of samples in controls to 50% in T2DM, and not by its measured values, which were unrelated to HbA1c where it was detected. Non-detection was concentrated in the samples affected by the peak-assignment inconsistency, and in the samples and profiles in which C17:0 was measured reliably the association was weaker and not significant. These subsets had limited power, and their confidence intervals include the full-sample estimate. The data therefore neither establish nor exclude an association, and resolving it requires quantification with greater sensitivity for minor components.

An inverse association of this kind would be consistent with prospective evidence. In the EPIC-InterAct case-cohort study, each standard deviation of plasma phospholipid C17:0 was associated with a hazard ratio for incident T2DM of 0.67 (95% CI 0.63–0.71) [26], and a pooled analysis of 16 prospective cohorts reported a hazard ratio per interdecile range of 0.65 (0.59–0.72) [27]; meta-analyses of circulating saturated fatty acids show the same contrast between even-chain and odd-chain species [28,29]. C17:0 reflects dairy fat intake but is also synthesized endogenously [30,31]. We cite this evidence as context for a question that the present data leave open, not as support for an association that they do not establish, and we do not interpret C17:0 biologically.

Taken together, higher monounsaturated proportions with higher triglycerides and a lower ratio of linoleic to palmitic acid with higher HbA1c are compatible with increased de novo lipogenesis and altered lipid handling in insulin resistance. Changes in lipid metabolism are also among those described in biological ageing [32], but neither biological age nor lipogenesis was measured, the design is cross-sectional, and reverse causation is equally compatible with the data. The present results do not establish a mechanistic link with biological ageing or any metabolic sequence.

### 3.5. Comparison with Kazakh and Neighboring Populations

To our knowledge, circulating fatty acid composition has not previously been reported for an ethnic Kazakh population; earlier metabolic studies in the parent cohort concerned serum amino acids [33] and the untargeted serum metabolome [34]. Indirect evidence is nonetheless consistent with the pattern observed here. Young adults in Kazakhstan report frequent consumption of meat and infrequent consumption of fish [35], and whole-blood samples from Kazakhstan in a large international survey showed low omega-3 status (mean omega-3 level 3.9%) and a high omega-6 to omega-3 ratio [36], although that sample was self-selected and its ethnicity was not recorded. In a comparison of Japanese, Korean and Mongolian workers, the Mongolian group had the lowest plasma eicosapentaenoic and docosahexaenoic acid levels and the highest insulin resistance [37]. Long-chain omega-3 fatty acids were below detection in most of our samples, so omega-3 status cannot be compared directly, and their non-detection should not be read as evidence of low levels. The prominence of associations with triglycerides in our data is consistent with the epidemiology of Kazakhs in neighbouring Xinjiang, among whom hypertriglyceridaemia and elevated LDL cholesterol were among the strongest correlates of diabetes in more than 118,000 adults [38]. In men from Western Siberia, plasma oleic acid was positively associated with a history of stroke and linoleic acid inversely with a history of myocardial infarction [39], which is directionally consistent with the associations of oleic acid with triglycerides and of linoleic acid with HbA1c observed here. These comparisons involve different matrices, lipid fractions and outcomes and indicate consistency of direction only.

### 3.6. Strengths and Limitations

The strengths of this study are the combined fatty acid and cardiometabolic phenotyping of an under-studied Central Asian population, control of the false discovery rate with exact q-values throughout, verification of every reported result from the source data with a single analysis script, and open reporting of the analytical problems found, with sensitivity analyses defined on biochemical grounds rather than by the associations observed.

The most important limitation is analytical. A sample-dependent inconsistency of peak assignment affected the C18 region: in 71 of the 177 profiles one peak was assigned to two components, stearic acid was implausibly high and minor components, including C17:0, were detected less often, and in most of the remaining profiles stearic acid was not recorded. The raw chromatograms could not be re-processed for this analysis. We identified implausible profiles by explicit criteria and report every principal result with and without them, but these criteria cannot exclude subtler misassignment, stearic acid could not be analysed at all, and the correction of the C18:1 artefact concerns the analytical validity of the composition, not the biological robustness of the associations that remain. The inconsistency was unrelated to glycemic group (χ^2^ *p* = 0.36) and to the clinical variables examined, so it is expected to dilute associations rather than to create them. Identification was based on retention time within fixed windows of the total ion current trace, with mass-spectral confirmation applied to the reference mixture and to a selection of samples only; repeatability was available for seven of the 13 components, and precision across the analytical series could not be reported.

Second, clinical variables other than FPG and HbA1c were not recorded for the T2DM group, and the reasons why variables were not recorded for particular participants were not documented. HbA1c was recorded to the nearest whole percentage point for most participants, and FPG values were not comparable between the two data subsets. All covariate-adjusted analyses are therefore restricted to 107 controls and participants with prediabetes, in whom HbA1c was effectively binary, and none of their results can be extended to the T2DM group.

Third, participants receiving glucose-lowering therapy were classified in the diabetes group (Section 4.2), so in every comparison involving that group the effect of treatment on fatty acid composition cannot be distinguished from that of diabetes itself. Lipid-lowering therapy and dietary intake were not recorded for any participant. None of the reported associations can therefore be regarded as independent of diet or treatment.

Fourth, fatty acids expressed as percentages are compositional data constrained to a constant sum, so correlations between individual proportions carry only relative information [40,41]; as shown in Section 2.6, the results of log-ratio analyses depend on the reference chosen. Fifth, the analysis is restricted to the 13 components detected in at least half of the samples, and it provides no information on long-chain polyunsaturated fatty acids, omega-3 status or pentadecanoic acid. Because detection depends on processing as well as on abundance, low detection should not be read as low circulating levels. The composition is relative, and absolute, fraction-specific quantification against isotope-labelled internal standards would be needed to extend the findings [42].

Sixth, the T2DM group comprised 18 participants, which is too small to support stable multivariable estimates; in such sparse-data settings maximum-likelihood estimates are biased away from the null [43,44], and the adjusted multinomial model could not be fitted at all. With 13 analytes and the effect sizes reported in large cohorts, the study was underpowered to detect group differences of the expected size, and the absence of significant differences should not be read as evidence of their absence. Finally, the design is cross-sectional, and no association reported here can be interpreted causally.

## 4. Materials and Methods

### 4.1. Study Design, Data Provenance and Analytical Sample

This cross-sectional study used archived serum samples from a previously established cohort recruited in Semey, Kazakhstan, whose recruitment and clinical and metabolomic characteristics have been described previously [34,45]. The present study addresses a distinct analytical question, the serum fatty acid composition and its associations with glycemic and cardiometabolic parameters.

The serum samples were transferred to the analytical laboratory together with an accompanying clinical dataset. The clinical data in that dataset were recorded by the examining physicians of the parent study, who entered the information available to them for each participant; no further clinical records were available, and the reasons why particular variables were not recorded for particular participants were not documented. Missing variables are therefore described as not recorded. The dataset identifies, for each participant, whether a complete clinical profile was obtained; the division is absolute, in that participants without a complete profile have no clinical variable other than FPG and HbA1c, and it corresponds to the presence of a recorded date of clinical examination.

The analytical sample was defined by two criteria, applied in this order. First, records with an identical sample identifier and identical values in all fields were retained once; this removed two records (both controls) and left 179 participants with archived serum. Second, samples in which no panel component was detected (Section 4.5) were excluded; this removed two samples (both prediabetes), in which sample preparation had failed. No other exclusion was made. The analytical sample comprised 177 participants (45 controls, 114 with prediabetes and 18 with T2DM). Differences in sample size between analyses reflect the availability of clinical variables only: 177 for analyses of FPG and HbA1c; 109 for analyses involving age, sex, serum lipids and creatinine; and 107 for analyses requiring BMI and waist circumference, including all covariate-adjusted models (Supplementary Figure S1 and Table S5).

### 4.2. Participant Classification

Participants were classified according to FPG, HbA1c and documented clinical history. Normoglycemia was defined as FPG < 5.6 mmol/L and HbA1c < 5.7%. Prediabetes was defined as FPG of 5.6–6.9 mmol/L and/or HbA1c of 5.7–6.4%. T2DM was defined as FPG ≥ 7.0 mmol/L and/or HbA1c ≥ 6.5%, or a previously established clinical diagnosis of diabetes. Participants receiving glucose-lowering therapy or with a documented history of diabetes were classified in the T2DM group regardless of their current biochemical values.

### 4.3. Clinical and Biochemical Measurements

Fasting venous blood samples were collected in the morning after an overnight fast of 8–12 h. Serum was separated by centrifugation at 3000 rpm for 10 min, aliquoted and stored at −80 °C without freeze–thaw cycles until analysis. FPG, HbA1c, total cholesterol, LDL cholesterol, HDL cholesterol and triglycerides were measured using validated clinical laboratory procedures. Anthropometric measurements included body weight, height, BMI and waist circumference. Systolic and diastolic blood pressure were measured according to standardized clinical procedures. HbA1c was recorded in the clinical dataset to the nearest whole percentage point for 117 of the 177 participants and to two decimal places for the remainder. FPG values were systematically lower in participants without a complete clinical profile than in those with one (Section 2.1), and the two sets of glucose measurements are therefore not regarded as directly comparable.

### 4.4. Serum Fatty Acid Analysis

Serum fatty acids were analyzed as fatty acid methyl esters (FAMEs) by gas chromatography–mass spectrometry (GC–MS). An aliquot of 100 µL of serum was subjected to alkaline hydrolysis and transesterification by adding 2 mL of 0.5 M potassium hydroxide in methanol and incubating at 70 °C for 15 min. Subsequently, 2 mL of 14% boron trifluoride in methanol (BF_3_–MeOH) was added and the samples were incubated at 70 °C for 30 min. After cooling, 2 mL of distilled water and 3 mL of n-hexane were added, the samples were thoroughly mixed, and the upper organic phase was collected and dried over anhydrous sodium sulfate. The resulting FAME extract was adjusted to a final volume of 1 mL with n-hexane prior to analysis. No internal standard was added.

GC–MS analysis was performed using a TRACE 1300 gas chromatograph coupled to a TSQ Series mass spectrometer (Thermo Fisher Scientific, Waltham, MA, USA) equipped with an AI/AS 1310 autosampler. Chromatographic separation was achieved on a TR-FAME capillary column (60 m × 0.25 mm i.d., 0.25 µm film thickness; Thermo Fisher Scientific). A 1.0 µL aliquot was injected through a programmed-temperature vaporizing (PTV) inlet operated in CT Split mode at 230 °C with a split ratio of 10:1 and a split flow of 15.0 mL/min. The carrier gas was operated in programmed-pressure mode. The oven temperature was initially 100 °C and was increased to 180 °C at 4 °C/min, then to 190 °C at 2 °C/min with a hold of 5 min, and finally to 250 °C at 2 °C/min with a final hold of 5 min. Mass spectrometric detection was performed in electron ionization mode with the ion source at 240 °C and the transfer line at 250 °C. Spectra were acquired in full-scan mode over *m*/*z* 30–550 with a scan time of approximately 0.2 s.

Data were processed in Thermo Xcalibur using the total ion current (TIC) trace. Each component was assigned to the highest peak within a 30 s identification window around its expected retention time, determined from a certified 37-component FAME reference mixture (Supelco 37 Component FAME Mix, CRM47885; Supelco, Bellefonte, PA, USA) analysed under identical conditions. No retention-time reference was used, and ion-ratio confirmation and spectral library matching were not enabled in the processing method. Mass spectra were used to confirm component identity in the reference mixture and were inspected manually for a selection of samples, but spectral confirmation was not applied systematically to every sample. Peaks were integrated with the ICIS algorithm without manual reintegration. For each component, Supplementary Table S1 gives the expected retention time, the identification window and the retention-time separation from the nearest neighboring component; the windows of adjacent components overlap in the C18 region.

All samples were analysed in a single analytical campaign with one sample-preparation protocol and one processing method, without column maintenance during the campaign. A pooled serum quality-control (QC) sample was analysed in five consecutive injections at the start of the analytical sequence and thereafter after every ten study samples. Intra-day repeatability from the five initial injections of independently prepared aliquots is reported for the seven components present at proportions sufficient for a meaningful coefficient of variation (coefficients of variation 1.19–5.95%; Supplementary Table S1b); in these injections the C18 region was assigned correctly (stearic acid 7.19%, oleic acid 16.40%). The results of the interspersed QC injections could not be retrieved, so precision across the whole series is not reported. Review of the injection sequence by the laboratory showed no systematic deviation in identification or integration related to injection order. Procedural blanks showed no fatty acid peaks above background. The instrument and processing parameters are documented in Supplementary Note S1.

### 4.5. Calibration, Composition, Detection and Peak-Assignment Checks

Sample values were obtained from a single-level external calibration for each component against the reference mixture at its nominal concentration (approximately 200, 400 or 600 µg/mL per component), with the regression forced through the origin. A 40-fold dilution series of the same mixture was analysed separately to characterize the linearity of response, and the limits of detection and quantification were derived from it as 3.3σ/S and 10σ/S, where σ is the standard deviation of the response and S the slope. Because these limits lie below the lowest calibration level, they are extrapolated estimates; they characterize the sensitivity of the method on the calibration standards and were not applied as thresholds to the sample values (Supplementary Table S1).

Relative composition. For each sample, the calibration-corrected amount of each of the 13 included components was divided by the summed amount of these components and expressed as a percentage. Reporting relative composition rather than absolute concentration is established practice for serum and plasma FAME analysis [46,47]. Because no internal standard was added before sample preparation, losses during extraction and the completeness of derivatization are unknown for each sample, and the amounts cannot be converted into concentrations in serum. The composition is therefore the percentage of the quantified profile, not of total serum fatty acid content, and no absolute concentrations are reported. For three samples the values supplied had already been scaled to a sum of 100; because the composition is invariant to scaling, this has no effect on the result.

Detection and inclusion. A component was recorded as detected in a sample when the ICIS algorithm found a peak with a signal-to-noise ratio of at least three within its identification window, and as zero otherwise. A component was included in the composition and in the statistical analysis if it was detected in at least 50% of samples; below this frequency the median and most of the distribution consist of non-detected values, and rank-based statistics are determined largely by the pattern of detection. Thirteen of the 37 panel components met this criterion. The criterion was defined by detection frequency and not by association with any outcome, and its effect was examined over thresholds from 25% to 70%.

Exclusion of C18:1n-9 trans. For every sample in which both C18:1 components were reported (n = 166), the ratio of the reported cis to trans values was computed. In 71–74 of these samples, depending on the tolerance applied (±0.2% to ±1%), the ratio was constant at 1.1298 (trans to cis, 0.8851), equal to the ratio of the single-level response factors of the two components. This is the signature of a single peak quantified twice against two calibrations, and the processing output for a representative sample confirmed an identical retention time and integrated response for both components. The same test applied to all 28 pairs of panel components eluting within one minute of each other found no other pair in which more than three samples shared a constant ratio within 0.2%. C18:1n-9 trans was therefore removed from the panel in every sample, and the composition was renormalized over the remaining components; all results were generated from this corrected dataset.

Peak-assignment inconsistency and plausibility criteria. The samples with the C18:1 duplication (71) also showed implausible values in the C18 region: relative to palmitic acid, stearic acid was recorded at about twice its proportion in the QC sample, exceeded oleic acid in 59% of samples and was strongly inversely related to it (ρ = −0.74), and minor components were detected less often. In 70 of the 95 samples in which both C18:1 components were reported without duplication, stearic acid was not recorded; C18:1n-9 trans was not reported in the remaining 11 samples. The laboratory confirmed that the processing configuration can assign one peak to several components and that a zero for stearic acid indicates the absence of a correctly computed result. The inconsistency was not related to glycemic group (χ^2^ *p* = 0.36), HbA1c, FPG, triglycerides, HDL cholesterol, BMI, age or data subset (all *p* ≥ 0.19); LDL cholesterol differed nominally (*p* = 0.041), which does not survive correction for the eight comparisons made. Stearic acid is therefore not interpreted. Profiles were classified as biochemically plausible if palmitic, oleic and linoleic acids were all detected, stearic acid did not exceed oleic acid, and linoleic acid did not exceed 60% of the panel; these criteria were set on biochemical grounds before the associations in the resulting subset were examined. In the 115 profiles meeting them, the ratios of minor components to palmitic acid agreed with those of the QC sample. Five alternative definitions were examined as sensitivity analyses. The full procedure is described in Supplementary Note S1.

### 4.6. Statistical Analysis

Continuous variables are summarized as median [interquartile range]. Between-group comparisons were performed with the Kruskal–Wallis test, followed where three groups could be compared by the Dunn post hoc test with Bonferroni correction for the three pairwise comparisons; epsilon-squared is reported as the effect size. Where only two groups had data, the Mann–Whitney test was used, with the Hodges–Lehmann estimate of the location shift and its 95% confidence interval. Categorical variables were compared with the chi-squared test.

Associations between fatty acids and clinical variables were assessed with the Spearman rank correlation coefficient. The Benjamini–Hochberg procedure [48] was applied separately within each clinical variable across the 13 fatty acids, since each clinical variable represents a distinct question; exact q-values are reported. Partial Spearman correlations were computed on the residuals of rank-transformed fatty acid proportions and clinical variables after linear regression on age, sex, BMI and waist circumference. Linear regression with standardized variables was used to estimate the association of each fatty acid with HbA1c and FPG, adjusted for age, sex and BMI, with one fatty acid per model and Benjamini–Hochberg correction within each outcome; the models were refitted with smoking status and antihypertensive medication added. Multinomial logistic regression adjusted for age, sex and BMI was planned but could not be estimated, because no covariate was recorded for any participant with T2DM.

Sensitivity analyses comprised: the comparison of all correlations before and after the C18:1 correction; analyses with a data subset as a covariate and within data subsets, including the participants without T2DM whose HbA1c was recorded as a continuous value; analyses in the samples affected and unaffected by the peak-assignment inconsistency, in the plausible profiles and under five alternative plausibility definitions; inclusion thresholds from 25% to 70%; exclusion of C4:0; and three log-ratio approaches [40], namely centred log-ratio transformation of all 13 components with multiplicative replacement of zeros [49] at 65% of the smallest detected value of each component, centred log-ratio transformation of the subcomposition of components detected in at least 85% of samples, and additive log-ratio to palmitic acid. For C17:0, censored (Tobit) regression [50] of log-transformed C17:0 on standardized HbA1c, with non-detected values left-censored at the smallest detected value, and logistic regression of detection on HbA1c were fitted; bootstrap 95% confidence intervals for Spearman coefficients were obtained from 5000 resamples, and power was calculated by Fisher’s z transformation. All tests were two-sided; *p* < 0.05 was considered nominally significant and q < 0.05 significant after correction. All results were generated from the source data by a single analysis script in Python (version 3.13) using SciPy, statsmodels and pandas.

## 5. Conclusions

In this exploratory cross-sectional study of 177 adults from Kazakhstan, serum fatty acid composition, expressed as percentages of the quantified profile of 13 components, was associated more consistently with triglycerides than with glycemic status. Oleic and palmitoleic acids were positively associated with triglycerides in controls and participants with prediabetes, and both associations persisted after adjustment for age, sex and adiposity and after exclusion of biochemically implausible profiles. Linoleic acid was inversely associated with HbA1c across all participants in unadjusted analyses. C17:0 was detected less often at higher HbA1c, and whether its proportion is associated with HbA1c is neither established nor excluded by these data.

The data do not show a fatty acid signature distinguishing normoglycemia, prediabetes and T2DM, a fatty acid profile independently associated with diabetes status, or a mechanistic link with biological ageing, and they provide no basis for the clinical use of any fatty acid as a marker. The findings rest on a cross-sectional design in which the covariate-adjusted analyses necessarily excluded the T2DM group, on HbA1c recorded with limited precision for most participants, on relative quantification, and on a dataset affected by a peak-assignment inconsistency in the C18 region. Confirmation will require re-processed or newly acquired chromatographic data, absolute and fraction-specific quantification against isotope-labelled internal standards, dietary and medication data, and a larger T2DM group with complete clinical phenotyping. Within these constraints, the study describes the serum fatty acid profile in this population and generates hypotheses for such studies.

## Figures and Tables

**Table 1:** Characteristics of participants by glycemic group.

Characteristic	Control	Prediabetes	T2DM	*n* (C/P/D)	*p*	ε^2^	Pairwise *p* or Shift [95% CI]
Male sex, *n/N*	5/24	25/85	n.a.	24/85/0	0.567	—	—
Age, years	49.00 [42.00–55.00]	53.00 [48.00–59.00]	n.a.	24/85/0	0.0284	0.036	+5.00 [+1.00; +9.00]
BMI, kg/m^2^	26.58 [23.23–28.80]	28.58 [25.20–31.25]	n.a.	24/83/0	0.025	0.038	+2.25 [+0.30; +4.46]
Waist circumference, cm	89.00 [82.00–94.00]	96.00 [89.00–105.00]	n.a.	24/83/0	0.00308	0.074	+8.00 [+3.00; +14.00]
Systolic BP, mmHg	115.00 [110.00–130.00]	120.00 [110.00–140.00]	n.a.	24/83/0	0.112	0.015	+10.00 [+0.00; +10.00]
Diastolic BP, mmHg	75.00 [70.00–82.50]	80.00 [70.00–90.00]	n.a.	24/83/0	0.0752	0.021	+10.00 [+0.00; +10.00]
Fasting glucose, mmol/L	4.70 [3.85–4.97]	5.21 [4.71–5.53]	7.43 [5.42–9.29]	45/114/18	3.4 × 10^−9^	0.213	C–P 9.4 × 10^−5^; C–D 4.8 × 10^−9^; P–D 5.4 × 10^−4^
HbA1c, %	5.00 [5.00–5.50]	6.00 [6.00–6.00]	7.26 [7.00–8.65]	45/114/18	3.9 × 10^−31^	0.793	C–P 4.4 × 10^−20^; C–D 2.1 × 10^−26^; P–D 2.6 × 10^−7^
Total cholesterol, mmol/L	4.75 [4.44–5.18]	5.38 [4.54–5.76]	n.a.	24/85/0	0.0109	0.051	+0.52 [+0.15; +0.87]
HDL cholesterol, mmol/L	1.48 [1.30–1.55]	1.24 [1.11–1.56]	n.a.	24/85/0	0.219	0.005	−0.11 [−0.27; +0.07]
LDL cholesterol, mmol/L	3.06 [2.76–3.34]	3.43 [2.90–3.99]	n.a.	24/85/0	0.0212	0.04	+0.39 [+0.06; +0.74]
Triglycerides, mmol/L	1.06 [0.78–1.22]	1.21 [0.93–1.59]	n.a.	24/85/0	0.0669	0.022	+0.18 [−0.01; +0.40]
Creatinine, µmol/L	69.00 [61.25–78.00]	66.00 [60.00–78.00]	n.a.	24/85/0	0.627	0.000	−2.00 [−8.00; +6.00]

Values are median [interquartile range] unless stated otherwise. *n* (C/P/D), number of participants contributing in the control, prediabetes and T2DM groups. FPG and HbA1c were compared across the three groups by the Kruskal–Wallis test followed by the Dunn test; pairwise values are Bonferroni-adjusted *p* (C, control; P, prediabetes; D, T2DM). All other variables were not recorded for the T2DM group (n.a.) and were compared between controls and prediabetes by the Mann–Whitney test, with the Hodges–Lehmann location shift (prediabetes minus control) and its 95% confidence interval; sex was compared by the chi-squared test. ε^2^, epsilon-squared effect size. No correction for multiple testing was applied in this table. HbA1c was recorded to the nearest whole percentage point for 117 of the 177 participants (105 of the 109 with a complete clinical profile), and FPG values are not directly comparable between participants with and without a complete clinical profile (Section 2.1). BMI, body mass index; BP, blood pressure; HbA1c, glycated hemoglobin; HDL, high-density lipoprotein; LDL, low-density lipoprotein; T2DM, type 2 diabetes mellitus.

**Table 2:** Serum fatty acid composition by glycemic status (percentage of the quantified profile).

A. All Participants						
Fatty acid	Control (*n* = 45)	Prediabetes (*n* = 114)	T2DM (*n* = 18)	H	*p*	q
C4:0 (butyric)	0.069 [0.056–0.085]	0.065 [0.053–0.094]	0.050 [0.041–0.071]	5.01	0.0815	0.177
C6:0 (caproic)	0.086 [0.000–0.344]	0.006 [0.000–0.132]	0.024 [0.000–0.262]	4.31	0.116	0.216
C8:0 (caprylic)	0.009 [0.000–0.014]	0.002 [0.000–0.010]	0.001 [0.000–0.006]	7.36	0.0253	0.100
C12:0 (lauric)	0.012 [0.004–0.017]	0.007 [0.000–0.014]	0.006 [0.001–0.010]	6.93	0.0313	0.100
C14:0 (myristic)	0.848 [0.572–1.118]	0.871 [0.266–1.104]	0.684 [0.442–1.102]	1.11	0.575	0.680
C16:0 (palmitic)	35.084 [31.548–37.877]	34.677 [32.303–37.878]	37.316 [35.417–39.107]	6.51	0.0385	0.100
C16:1 (palmitoleic)	0.848 [0.484–1.424]	1.063 [0.411–1.727]	1.117 [0.177–1.838]	0.51	0.775	0.781
C17:0 (heptadecanoic)	0.254 [0.052–0.349]	0.187 [0.000–0.332]	0.012 [0.000–0.195]	7.64	0.0219	0.100
C18:0 (stearic) †	0.000 [0.000–15.484]	13.343 [0.000–16.903]	7.533 [0.000–19.736]	1.94	0.379	0.547
C18:1n-9 (oleic)	15.808 [13.579–17.478]	15.788 [13.774–18.421]	16.312 [12.115–19.042]	0.49	0.781	0.781
C18:2n-6 (linoleic)	35.899 [31.835–39.698]	33.139 [29.145–37.473]	31.915 [27.577–34.869]	7.94	0.0189	0.100
C20:3n-6 (DGLA)	3.082 [2.040–4.532]	3.034 [1.593–3.964]	2.874 [1.075–4.761]	2.17	0.339	0.547
C24:0 (lignoceric)	0.163 [0.000–0.311]	0.106 [0.000–0.279]	0.042 [0.000–0.266]	1.57	0.457	0.594
B. Biochemically plausible profiles						
Fatty acid	Control (*n* = 30)	Prediabetes (*n* = 74)	T2DM (*n* = 11)	H	*p*	q
C4:0 (butyric)	0.067 [0.053–0.082]	0.064 [0.055–0.091]	0.051 [0.044–0.070]	3.55	0.169	0.411
C6:0 (caproic)	0.094 [0.000–0.357]	0.012 [0.000–0.201]	0.136 [0.024–0.287]	3.32	0.190	0.411
C8:0 (caprylic)	0.008 [0.000–0.014]	0.003 [0.000–0.010]	0.003 [0.001–0.011]	2.08	0.354	0.568
C12:0 (lauric)	0.012 [0.002–0.017]	0.007 [0.002–0.014]	0.009 [0.004–0.014]	2.89	0.236	0.438
C14:0 (myristic)	0.916 [0.667–1.194]	0.971 [0.710–1.189]	0.936 [0.753–1.282]	0.41	0.813	0.813
C16:0 (palmitic)	36.194 [31.613–38.550]	35.078 [32.586–37.923]	38.060 [36.168–39.264]	3.66	0.160	0.411
C16:1 (palmitoleic)	1.294 [0.740–1.846]	1.304 [0.888–1.968]	1.791 [1.386–2.035]	1.71	0.425	0.568
C17:0 (heptadecanoic)	0.264 [0.069–0.374]	0.229 [0.045–0.365]	0.156 [0.036–0.312]	1.29	0.526	0.622
C18:0 (stearic) †	0.000 [0.000–8.683]	0.000 [0.000–14.298]	0.000 [0.000–0.000]	5.47	0.0648	0.411
C18:1n-9 (oleic)	16.546 [14.715–17.976]	17.307 [15.678–19.050]	18.517 [16.312–22.312]	3.59	0.167	0.411
C18:2n-6 (linoleic)	37.720 [35.244–40.396]	34.576 [30.876–38.458]	34.090 [31.152–35.592]	9.28	0.00966	0.126
C20:3n-6 (DGLA)	3.168 [2.566–4.372]	3.168 [2.010–4.002]	3.417 [2.373–4.945]	0.72	0.697	0.755
C24:0 (lignoceric)	0.238 [0.063–0.365]	0.158 [0.007–0.311]	0.252 [0.147–0.319]	1.66	0.437	0.568

Values are median [interquartile range] of the percentage of the summed amount of the 13 quantified components; non-detected values enter as zero. Groups were compared by the Kruskal–Wallis test (H, test statistic); q, Benjamini–Hochberg false discovery rate-adjusted *p* across the 13 components within each panel. Comparisons involving the T2DM group are descriptive and are not adjusted for age, sex, adiposity or treatment. Panel (B) is restricted to the profiles meeting the plausibility criteria in Section 4.5. † Stearic acid values are affected by the peak-assignment inconsistency in the C18 region and are not interpretable (Section 4.5). C4:0, butyric (butanoic) acid; C6:0, caproic (hexanoic) acid; C8:0, caprylic (octanoic) acid; C12:0, lauric (dodecanoic) acid; C14:0, myristic (tetradecanoic) acid; C16:0, palmitic (hexadecanoic) acid; C16:1, palmitoleic (cis-9-hexadecenoic) acid; C17:0, heptadecanoic (margaric) acid; C18:0, stearic (octadecanoic) acid; C18:1n-9, oleic (cis-9-octadecenoic) acid; C18:2n-6, linoleic (cis,cis-9,12-octadecadienoic) acid; C20:3n-6, dihomo-γ-linolenic (cis-8,11,14-eicosatrienoic) acid (DGLA); C24:0, lignoceric (tetracosanoic) acid.

**Table 3:** Pairwise comparisons for fatty acids with nominal between-group differences (all participants; *n* = 45/114/18).

Fatty Acid	Kruskal–Wallis *p*	Control vs. Prediabetes	Control vs. T2DM	Prediabetes vs. T2DM
C8:0 (caprylic)	0.0253	0.044	0.092	1.000
C12:0 (lauric)	0.0313	0.048	0.126	1.000
C16:0 (palmitic)	0.0385	1.000	0.041	0.058
C17:0 (heptadecanoic)	0.0219	0.270	0.020	0.212
C18:2n-6 (linoleic)	0.0189	0.132	0.023	0.373

Pairwise values are Bonferroni-adjusted *p*-values from the Dunn test (three comparisons); they are not false discovery rate q-values. None of the Kruskal–Wallis *p*-values remained significant after Benjamini–Hochberg correction across the 13 components (Table 2). In the plausible profiles only linoleic acid differed nominally (Kruskal–Wallis *p* = 0.0097; control vs. prediabetes *p* = 0.019; control vs. T2DM *p* = 0.049; prediabetes vs. T2DM *p* = 1.000). Comparisons with the T2DM group are descriptive. C8:0, caprylic (octanoic) acid; C12:0, lauric (dodecanoic) acid; C16:0, palmitic (hexadecanoic) acid; C17:0, heptadecanoic (margaric) acid; C18:2n-6, linoleic (cis,cis-9,12-octadecadienoic) acid.

**Table 4:** Unadjusted Spearman correlations between serum fatty acids and glycemic and cardiometabolic parameters, ρ (q).

Fatty Acid	HbA1c (*n* = 177)	Fasting Glucose (*n* = 177)	BMI (*n* = 107)	Triglycerides (*n* = 109)	HDL-C (*n* = 109)	LDL-C (*n* = 109)
C4:0 (butyric)	−0.062 (0.485)	−0.049 (0.596)	−0.022 (0.822)	−0.287 (0.011)	−0.078 (0.621)	−0.212 (0.071)
C6:0 (caproic)	−0.102 (0.293)	−0.024 (0.755)	−0.072 (0.679)	−0.126 (0.278)	+0.160 (0.235)	+0.112 (0.321)
C8:0 (caprylic)	−0.183 (0.063)	−0.122 (0.230)	−0.146 (0.348)	−0.104 (0.364)	+0.069 (0.621)	+0.073 (0.485)
C12:0 (lauric)	−0.146 (0.138)	−0.141 (0.230)	−0.071 (0.679)	−0.169 (0.147)	+0.155 (0.235)	+0.029 (0.764)
C14:0 (myristic)	−0.092 (0.293)	+0.045 (0.596)	−0.057 (0.731)	+0.192 (0.120)	+0.010 (0.915)	+0.101 (0.348)
C16:0 (palmitic)	+0.174 (0.066)	+0.184 (0.093)	+0.080 (0.679)	−0.177 (0.141)	−0.026 (0.915)	−0.223 (0.071)
C16:1 (palmitoleic)	+0.034 (0.656)	+0.080 (0.455)	+0.155 (0.348)	+0.319 (0.005)	+0.080 (0.621)	+0.162 (0.154)
C17:0 (heptadecanoic)	−0.218 (0.033)	−0.117 (0.230)	−0.152 (0.348)	+0.040 (0.800)	+0.076 (0.621)	+0.150 (0.174)
C18:0 (stearic) †	+0.095 (0.293)	−0.121 (0.230)	−0.030 (0.818)	−0.236 (0.044)	−0.013 (0.915)	−0.279 (0.021)
C18:1n-9 (oleic)	+0.041 (0.635)	+0.209 (0.069)	+0.291 (0.030)	+0.647 (<0.001)	−0.258 (0.088)	+0.215 (0.071)
C18:2n-6 (linoleic)	−0.210 (0.033)	−0.076 (0.455)	−0.091 (0.679)	+0.000 (1.000)	+0.164 (0.235)	+0.335 (0.005)
C20:3n-6 (DGLA)	−0.111 (0.293)	−0.116 (0.230)	−0.186 (0.348)	+0.008 (1.000)	+0.205 (0.210)	+0.161 (0.154)
C24:0 (lignoceric)	−0.098 (0.293)	−0.068 (0.476)	−0.040 (0.802)	+0.128 (0.278)	+0.164 (0.235)	+0.179 (0.136)

Fatty acids are expressed as percentages of the quantified profile. Each cell gives the Spearman correlation coefficient ρ and, in parentheses, the Benjamini–Hochberg false discovery rate-adjusted q-value within that clinical parameter across the 13 fatty acids; exact *p*-values are given in Supplementary Table S7. n, number of participants contributing to each column. The HbA1c and FPG columns include all three glycemic groups; the BMI and lipid columns include controls and participants with prediabetes only, because these variables were not recorded for the T2DM group. Unadjusted. † Not interpretable (Section 4.5). HDL-C, HDL cholesterol; LDL-C, LDL cholesterol. C4:0, butyric (butanoic) acid; C6:0, caproic (hexanoic) acid; C8:0, caprylic (octanoic) acid; C12:0, lauric (dodecanoic) acid; C14:0, myristic (tetradecanoic) acid; C16:0, palmitic (hexadecanoic) acid; C16:1, palmitoleic (cis-9-hexadecenoic) acid; C17:0, heptadecanoic (margaric) acid; C18:0, stearic (octadecanoic) acid; C18:1n-9, oleic (cis-9-octadecenoic) acid; C18:2n-6, linoleic (cis,cis-9,12-octadecadienoic) acid; C20:3n-6, dihomo-γ-linolenic (cis-8,11,14-eicosatrienoic) acid (DGLA); C24:0, lignoceric (tetracosanoic) acid.

**Table 5:** Partial Spearman correlations adjusted for age, sex, body mass index and waist circumference (controls and prediabetes).

Clinical Parameter	Fatty Acid	Partial r (q), All with Covariates (*n* = 107)	Partial r (q), Plausible Profiles (*n* = 71)
Triglycerides	C18:1n-9 (oleic)	+0.612 (<0.001)	+0.735 (<0.001)
Triglycerides	C16:1 (palmitoleic)	+0.247 (0.027)	+0.290 (0.037)
Triglycerides	C16:0 (palmitic)	−0.260 (0.022)	−0.331 (0.026)
Triglycerides	C4:0 (butyric)	−0.313 (0.007)	−0.280 (0.039)
Triglycerides	C6:0 (caproic)	−0.103 (0.418)	−0.315 (0.026)
Triglycerides	C12:0 (lauric)	−0.175 (0.155)	−0.272 (0.041)
Triglycerides	C18:2n-6 (linoleic)	+0.082 (0.509)	−0.313 (0.026)
Triglycerides	C18:0 (stearic) †	−0.274 (0.018)	+0.078 (0.670)
HDL-C	C18:1n-9 (oleic)	−0.242 (0.157)	−0.357 (0.029)
LDL-C	C18:2n-6 (linoleic)	+0.389 (<0.001)	+0.347 (0.039)
LDL-C	C18:0 (stearic) †	−0.261 (0.043)	−0.054 (0.795)
HbA1c	C17:0 (heptadecanoic)	−0.100 (0.751)	−0.097 (0.729)
HbA1c	C18:2n-6 (linoleic)	+0.029 (0.827)	−0.013 (0.915)

Fatty acids are expressed as percentages of the quantified profile. Partial Spearman correlation coefficients (r) computed on the residuals of rank-transformed fatty acid proportions and clinical parameters after linear regression on age, sex, BMI and waist circumference. q, Benjamini–Hochberg false discovery rate-adjusted *p* within each clinical parameter across the 13 fatty acids; exact *p*-values are given in Supplementary Table S7. Rows show every association with q < 0.05 in either column, and the two HbA1c associations discussed in the text. No participant with T2DM is included, because the covariates were not recorded for that group. † Not interpretable (Section 4.5). C4:0, butyric (butanoic) acid; C6:0, caproic (hexanoic) acid; C12:0, lauric (dodecanoic) acid; C16:0, palmitic (hexadecanoic) acid; C16:1, palmitoleic (cis-9-hexadecenoic) acid; C17:0, heptadecanoic (margaric) acid; C18:0, stearic (octadecanoic) acid; C18:1n-9, oleic (cis-9-octadecenoic) acid; C18:2n-6, linoleic (cis,cis-9,12-octadecadienoic) acid.

**Table 6:** Linear regression of HbA1c and fasting plasma glucose on each serum fatty acid, adjusted for age, sex and body mass index (controls and prediabetes; *n* = 107).

Fatty Acid	HbA1c: β [95% CI]	*P*	q	Fasting Glucose: β [95% CI]	*p*	q
C4:0 (butyric)	−0.009 [−0.204; +0.186]	0.924	0.938	+0.150 [−0.043; +0.342]	0.126	0.813
C6:0 (caproic)	+0.008 [−0.189; +0.204]	0.938	0.938	+0.022 [−0.174; +0.218]	0.827	0.909
C8:0 (caprylic)	−0.198 [−0.386; −0.010]	0.039	0.344	−0.093 [−0.284; +0.097]	0.333	0.813
C12:0 (lauric)	−0.168 [−0.356; +0.020]	0.079	0.344	−0.101 [−0.290; +0.089]	0.296	0.813
C14:0 (myristic)	−0.134 [−0.323; +0.055]	0.163	0.354	+0.030 [−0.161; +0.220]	0.759	0.909
C16:0 (palmitic)	−0.086 [−0.277; +0.104]	0.370	0.687	−0.019 [−0.209; +0.172]	0.846	0.909
C16:1 (palmitoleic)	+0.017 [−0.177; +0.212]	0.859	0.938	+0.125 [−0.068; +0.318]	0.201	0.813
C17:0 (heptadecanoic)	−0.139 [−0.329; +0.052]	0.152	0.354	−0.011 [−0.203; +0.181]	0.909	0.909
C18:0 (stearic) †	+0.075 [−0.116; +0.266]	0.438	0.712	+0.034 [−0.157; +0.225]	0.727	0.909
C18:1n-9 (oleic)	+0.048 [−0.146; +0.241]	0.627	0.905	+0.167 [−0.024; +0.357]	0.086	0.813
C18:2n-6 (linoleic)	+0.017 [−0.174; +0.208]	0.859	0.938	−0.085 [−0.276; +0.105]	0.375	0.813
C20:3n-6 (DGLA)	−0.142 [−0.333; +0.050]	0.146	0.354	−0.043 [−0.236; +0.151]	0.663	0.909
C24:0 (lignoceric)	−0.188 [−0.378; +0.002]	0.053	0.344	−0.041 [−0.234; +0.152]	0.674	0.909

Fatty acids are expressed as percentages of the quantified profile. Each row is a separate model containing one fatty acid together with age, sex and BMI. β, standardized regression coefficient (change in the outcome, in standard deviations, per 1-SD increase in the fatty acid proportion) with its 95% confidence interval; q, Benjamini–Hochberg false discovery rate-adjusted *p* within each outcome across the 13 fatty acids. In this subset HbA1c was 5.0% or 6.0% for 102 of the 107 participants. No participant with T2DM is included. † Not interpretable (Section 4.5). C4:0, butyric (butanoic) acid; C6:0, caproic (hexanoic) acid; C8:0, caprylic (octanoic) acid; C12:0, lauric (dodecanoic) acid; C14:0, myristic (tetradecanoic) acid; C16:0, palmitic (hexadecanoic) acid; C16:1, palmitoleic (cis-9-hexadecenoic) acid; C17:0, heptadecanoic (margaric) acid; C18:0, stearic (octadecanoic) acid; C18:1n-9, oleic (cis-9-octadecenoic) acid; C18:2n-6, linoleic (cis,cis-9,12-octadecadienoic) acid; C20:3n-6, dihomo-γ-linolenic (cis-8,11,14-eicosatrienoic) acid (DGLA); C24:0, lignoceric (tetracosanoic) acid.

**Table 7:** Summary of the evidence for the principal associations, by population and analysis.

Association	Analysis	Population (*n*)	Estimate	q	Assessment
Oleic acid × triglycerides	Unadjusted	Controls and prediabetes (109)	ρ = +0.647	<0.001	Supported in every analysis
	Unadjusted, plausible profiles	Controls and prediabetes (72)	ρ = +0.742	<0.001	
	Partial, adjusted	Controls and prediabetes (107)	r = +0.612	<0.001	
	Partial, adjusted, plausible profiles	Controls and prediabetes (71)	r = +0.735	<0.001	
Palmitoleic acid × triglycerides	Unadjusted	Controls and prediabetes (109)	ρ = +0.319	0.005	Supported; also under all log-ratio approaches
	Unadjusted, plausible profiles	Controls and prediabetes (72)	ρ = +0.332	0.014	
	Partial, adjusted	Controls and prediabetes (107)	r = +0.247	0.027	
	Partial, adjusted, plausible profiles	Controls and prediabetes (71)	r = +0.290	0.037	
Linoleic acid × HbA1c	Unadjusted	All participants (177)	ρ = −0.210	0.033	Unadjusted only; borderline;
	Unadjusted, plausible profiles	All participants (115)	ρ = −0.272	0.042	
	Regression, adjusted	Controls and prediabetes (107)	β = +0.017	0.938	
Linoleic acid × LDL cholesterol	Unadjusted	Controls and prediabetes (109)	ρ = +0.335	0.005	Not robust; inconsistent between processing subsets
	Unadjusted, plausible profiles	Controls and prediabetes (72)	ρ = +0.283	0.206	
	Partial, adjusted	Controls and prediabetes (107)	r = +0.389	<0.001	
Oleic acid × BMI	Unadjusted	Controls and prediabetes (107)	ρ = +0.291	0.030	Not robust
	Unadjusted, plausible profiles	Controls and prediabetes (71)	ρ = +0.265	0.173	
C17:0 × HbA1c	Unadjusted	All participants (177)	ρ = −0.218	0.033	Neither established nor excluded; depends on non-detection
	Unadjusted, plausible profiles	All participants (115)	ρ = −0.161	0.416	
	Regression, adjusted	Controls and prediabetes (107)	β = −0.139	0.354	
	Tobit, unaffected samples	All participants (95)	β = −0.028 (*p* = 0.84)	—	
Any fatty acid × glycemic group	Kruskal–Wallis	All participants (177)	—	≥0.100	No difference after correction
	Kruskal–Wallis, plausible profiles	All participants (115)	—	≥0.126	

Fatty acids are expressed as percentages of the quantified profile. “All participants” includes the three glycemic groups; “controls and prediabetes” excludes the T2DM group, for which lipids, anthropometry and covariates were not recorded. Adjusted analyses include age, sex and BMI; partial correlations are additionally adjusted for waist circumference. ρ, Spearman correlation coefficient; r, partial Spearman correlation coefficient; β, standardized regression coefficient (per 1-SD increase in the fatty acid for linear regression; per 1-SD increase in HbA1c for Tobit regression of log C17:0). q, Benjamini–Hochberg false discovery rate-adjusted *p* within each clinical parameter across the 13 fatty acids; —, not applicable. Plausible profiles, profiles meeting the biochemical plausibility criteria in Section 4.5; unaffected samples, samples without the C18:1 duplication. Full results of all sensitivity analyses are in Supplementary Table S6.

## Data Availability

The original contributions presented in this study are included in the article/Supplementary Materials. Further inquiries can be directed to the corresponding authors.

## Supplementary Materials

The following supporting information can be downloaded at: https://www.mdpi.com/article/10.3390/ijms27219702/s1.

## Abbreviations

The following abbreviations are used in this manuscript:

ALR	Additive log-ratio
BMI	Body mass index
CI	Confidence interval
clr	Centred log-ratio
CRM	Certified reference material
DGLA	Dihomo-γ-linolenic acid
FAME	Fatty acid methyl ester
FDR	False discovery rate
FPG	Fasting plasma glucose
GC–MS	Gas chromatography–mass spectrometry
HbA1c	Glycated hemoglobin
IQR	Interquartile range
LOD	Limit of detection
LOQ	Limit of quantification
T2DM	Type 2 diabetes mellitus
TIC	Total ion current
