# Supplementary material for: Serum Fatty Acid Profiles Across Normoglycemia, Prediabetes, and Type 2 Diabetes: Associations with Glycemic and Cardiometabolic Parameters in a Kazakh Population
Source: Int J Mol Sci. 2026 Oct 29;27(21):9702. doi: 10.3390/ijms27219702 (PMC13763187; doi:10.3390/ijms27219702)
Supplement: Supplementary file 1 [file ijms-27-09702-s001.zip › ijms-4600973-supplementary.pdf]

Supplementary Materials

Serum Fatty Acid Profiles across Normoglycemia, Prediabetes, and Type 2 Diabetes: Associations with Glycemic and Cardiometabolic Parameters in a Kazakh Population

Manuscript ID: ijms-4600973

Contents. Figure S1. Participant flow. Note S1. Analytical procedure, identification and correction of the C18:1 artefact, peak-assignment inconsistency and plausibility criteria. Table S1. Analytical characteristics of the 37 panel components (A, identification; B, calibration and detection). Table S1b. Intra-day repeatability in the pooled serum quality-control sample. Table S2. Detection frequency and group comparisons for all 37 panel components. Table S3. Partial Spearman correlations, full matrix. Table S4. Correlations and group comparisons before and after the C18:1 correction. Table S5. Availability of clinical variables by glycemic group. Table S6. Sensitivity analyses. Table S7. Exact p-values for the correlation analyses.

Figure S1. Participant flow

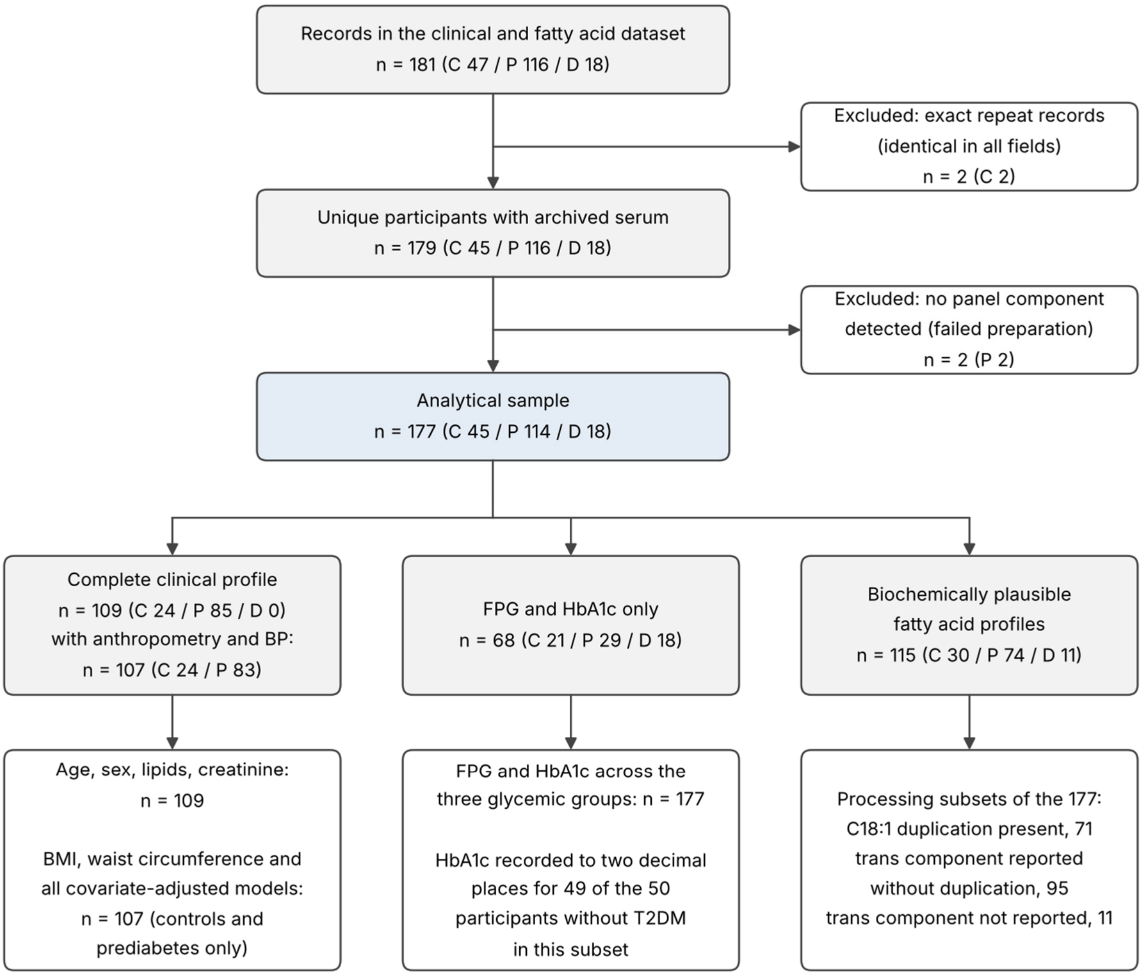

Figure S1. Construction of the analytical sample and of the subsets used in the analyses. Exact repeat records had an identical sample identifier and identical values in every clinical and fatty acid field and were retained once. No clinical variable other than fasting plasma glucose (FPG) and glycated hemoglobin (HbA1c) was recorded for any participant with T2DM. The plausible profiles and the processing subsets are defined in Note S1. BP, blood pressure; BMI, body mass index.

Note S1. Analytical procedure and data checks

S1.1. Instrument and processing parameters

Serum fatty acids were determined as fatty acid methyl esters on a TRACE 1300 gas chromatograph coupled to a TSQ series mass spectrometer (Thermo Fisher Scientific) with a TR-FAME column (60 m × 0.25 mm × 0.25 μm), electron ionization and full-scan acquisition (m/z 30–550); sample preparation and chromatographic conditions are given in Section 4.4 of the main text. Data were processed in Thermo Xcalibur with an external-standard processing method in which every component was quantified on the total ion current (TIC) trace. The parameters applied to all 37 components were: peak detection by the ICIS algorithm (smoothing 7 points, baseline window 40 scans, area and peak noise factors 10, minimum peak height S/N 3.0); no retention-time reference and no adjustment of expected retention times; ion-ratio confirmation and spectral library matching disabled; response measured as peak area; single calibration level; linear calibration forced through the origin; equal weighting; units μg/mL ("mcg/ml" in the method). The identification window was 30 s for every component except C4:0 (10 s). Thirty-one components were assigned to the highest peak within their window and six (C18:1n-9 trans, C18:3n-6, C21:0, C20:2n-6, C20:3n-6 and C22:1n-9) to the peak nearest to the expected retention time (Table S1A). In the processing method, C20:3n-6 (dihomo-γ-linolenic acid, cis-8,11,14) is labelled "C20:3_n3_cis_8,11,14"; the label refers to the n-6 isomer, as its double-bond positions show.

S1.2. Calibration, limits and composition

Sample values were obtained from a single-level calibration of each component against the certified 37-component FAME reference mixture (Supelco 37 Component FAME Mix, CRM47885) at its nominal concentration (202–612 μg/mL; Table S1B), forced through the origin. A separate 40-fold dilution series of the same mixture was used only to characterise linearity and to estimate the limits of detection and quantification (LOD = 3.3σ/S, LOQ = 10σ/S; median LOQ/LOD ratio 3.04). These limits lie at about 4% of the lowest calibration level and are therefore extrapolated; they were not applied to the sample values. In the previous version the units of the calibration range and of the limits were given as ng/mL; the correct unit is μg/mL, since the upper end of every calibration range equals the calibration level of the corresponding component in the processing method (for two components, C15:1 and C24:0, the range reported in the laboratory linearity table ends at 203 and 204 μg/mL whereas the method level is 204 and 404 μg/mL; the method values are shown). The composition of each sample is the calibration-corrected amount of each of the 13 included components divided by their sum, × 100. No internal standard was added, so the result is relative. For three samples the values supplied had already been scaled to a sum of 100, which has no effect on the composition.

S1.3. Detection and inclusion

A component was recorded as detected when a peak with S/N ≥ 3 was found within its window and as zero otherwise; no quantitation limit was applied to the sample values. Components detected in at least 50% of samples (13 of 37) were included. These 13 components account for a median of 99.9% (interquartile range 99.4–100.0%; minimum 97.0%) of the summed values of all detected panel components other than C18:1n-9 trans. Detection depends on processing as well as on abundance (S1.5).

S1.4. Identification and correction of the C18:1 artefact

C18:1n-9 trans was reported in 166 of the 177 samples, and both C18:1 components in 164. For each of the latter, the ratio of the reported cis to trans values was computed. In 71, 73 and 74 samples (tolerance ±0.2%, ±0.5% and ±1%) the ratio was constant at 1.1298 (trans to cis 0.8851), identical to at least six significant figures within the ±0.2% cluster over a 73-fold range of values. This constant equals the ratio of the single-level response factors of the two components (557107/493120 = 1.12976) and differs from the ratio of the slopes of the multi-level curves (1.0034), which shows that a single peak was quantified twice against the two single-level calibrations; the processing output for a representative sample showed an identical retention time and integrated area for both components. C18:1n-9 trans was identified by the nearest-retention-time rule, and the cis peak elutes 19.2 s after its expected retention time, which is consistent with this mechanism. The same test applied to all 28 pairs of

panel components eluting within one minute of each other found no other pair in which more than three samples shared a constant ratio within ±0.2%. C18:1n-9 trans was removed from the panel in every sample, and the composition was renormalized over the remaining components; the effect of the correction on every correlation and group comparison is shown in Table S4.

S1.5. Peak-assignment inconsistency in the C18 region and plausibility criteria

Samples were classified into three processing subsets: duplication present (n = 71); trans component reported without duplication (n = 95); trans component not reported (n = 11). In the samples with the duplication, stearic acid relative to palmitic acid was about twice its proportion in the pooled quality-control (QC) sample, exceeded oleic acid in 59% of samples and was strongly inversely related to it ($\varrho = -0.74$), and minor components, including C17:0, were detected less often (Table S6E). In 68 of the 95 samples in which the trans component was reported without duplication, stearic acid was not recorded. Adjacent components in the C18 region elute 19–26 s apart (Table S1A), within the 30 s identification window. The laboratory confirmed that this configuration can assign one peak to several components and that a zero for stearic acid indicates that no correct result was computed; in the five QC injections at the start of the sequence the C18 region was assigned correctly, and no relation to injection order was found. The processing subset was not related to glycemic group or to the clinical variables examined (Table S6G).

Profiles were classified as biochemically plausible when (1) palmitic, oleic and linoleic acids were all detected; (2) stearic acid did not exceed oleic acid; and (3) linoleic acid did not exceed 60% of the panel. The criteria were set on biochemical grounds, without reference to the associations. 115 profiles met them (30 / 74 / 11 in C / P / D; 29 of the 71 samples with the duplication and 77 of the 95 without it). Five alternative definitions were examined (Table S6A). Stearic acid is not interpreted in any analysis.

S1.6. Repeatability and quality control

A pooled serum QC sample was injected five times at the start of the analytical sequence and thereafter after every ten study samples. Intra-day repeatability from the five initial injections is given in Table S1b for the seven components present at sufficient proportions. The results of the interspersed QC injections could not be retrieved, and repeatability for the remaining six included components was not available; precision across the whole series therefore cannot be reported. Procedural blanks showed no fatty acid peaks above background. All samples were stored at −80 °C without freeze–thaw cycles and analysed in a single campaign with one preparation protocol and one processing method, without manual reintegration.

Table S1. Analytical characteristics of the 37 panel components, in order of elution. **A.** Identification.

No.	Component	Common name	Systematic name (methyl ester analysed)	Expected RT, min	Identification rule	Nearest component (Δ, s)
1	C4:0	Butyric	Butanoic	5.67	Highest peak	C6:0 (60.0)
2	C6:0	Caproic	Hexanoic	6.67	Highest peak	C4:0 (60.0)
3	C8:0	Caprylic	Octanoic	8.54	Highest peak	C6:0 (112.2)
4	C10:0	Capric	Decanoic	11.37	Highest peak	C11:0 (99.6)
5	C11:0	Undecylic	Undecanoic	13.03	Highest peak	C10:0 (99.6)
6	C12:0	Lauric	Dodecanoic	14.79	Highest peak	C11:0 (105.6)
7	C13:0	Tridecylic	Tridecanoic	16.56	Highest peak	C12:0 (106.2)
8	C14:0	Myristic	Tetradecanoic	18.34	Highest peak	C14:1 (58.2)
9	C14:1	Myristoleic	cis-9-Tetradecenoic	19.31	Highest peak	C15:0 (45.6)
10	C15:0	Pentadecylic	Pentadecanoic	20.07	Highest peak	C14:1 (45.6)
11	C15:1	—	cis-10-Pentadecenoic	21.06	Highest peak	C16:0 (47.4)
12	C16:0	Palmitic	Hexadecanoic	21.85	Highest peak	C16:1 (46.8)
13	C16:1	Palmitoleic	cis-9-Hexadecenoic	22.63	Highest peak	C16:0 (46.8)
14	C17:0	Margaric (heptadecanoic)	Heptadecanoic	23.63	Highest peak	C17:1 (51.0)
15	C17:1	—	cis-10-Heptadecenoic	24.48	Highest peak	C17:0 (51.0)
16	C18:0	Stearic	Octadecanoic	25.52	Highest peak	C18:1n-9 trans (25.8)
17	C18:1n-9 trans	Elaidic	trans-9-Octadecenoic	25.95	Nearest RT	C18:1n-9 (19.2)
18	C18:1n-9	Oleic	cis-9-Octadecenoic	26.27	Highest peak	C18:1n-9 trans (19.2)
19	C18:2n-6 trans	Linolelaidic	trans,trans-9,12-Octadecadienoic	26.82	Highest peak	C18:1n-9 (33.0)
20	C18:2n-6	Linoleic	cis,cis-9,12-Octadecadienoic	27.58	Highest peak	C18:2n-6 trans (45.6)
21	C20:0	Arachidic	Eicosanoic	28.50	Highest peak	C18:3n-6 (46.8)
22	C18:3n-6	γ-Linolenic	cis-6,9,12-Octadecatrienoic	29.28	Nearest RT	C18:3n-3 (20.4)
23	C18:3n-3	α-Linolenic	cis-9,12,15-Octadecatrienoic	29.62	Highest peak	C18:3n-6 (20.4)
24	C20:1n-9	Gondoic	cis-11-Eicosenoic	30.47	Highest peak	C18:3n-3 (51.0)
25	C21:0	Heneicosylic	Heneicosanoic	31.84	Nearest RT	C20:2n-6 (10.8)
26	C20:2n-6	Eicosadienoic	cis-11,14-Eicosadienoic	32.02	Nearest RT	C21:0 (10.8)
27	C22:0	Behenic	Docosanoic	33.05	Highest peak	C20:3n-6 (43.2)
28	C20:3n-6	Dihomo-γ-linolenic (DGLA)	cis-8,11,14-Eicosatrienoic	33.77	Nearest RT	C22:1n-9 (9.0)
29	C22:1n-9	Erucic	cis-13-Docosenoic	33.92	Nearest RT	C20:3n-6 (9.0)
30	C20:3n-3	Eicosatrienoic	cis-11,14,17-Eicosatrienoic	34.21	Highest peak	C22:1n-9 (17.4)
31	C23:0	Tricosylic	Tricosanoic	35.15	Highest peak	C20:4n-6 (38.4)
32	C20:4n-6	Arachidonic	cis-5,8,11,14-Eicosatetraenoic	35.79	Highest peak	C23:0 (38.4)

No.	Component	Common name	Systematic name (methyl ester analysed)	Expected RT, min	Identification rule	Nearest component (Δ, s)
33	C22:2n-6	Docosadienoic	cis-13,16-Docosadienoic	36.57	Highest peak	C24:0 (15.6)
34	C24:0	Lignoceric	Tetracosanoic	36.83	Highest peak	C22:2n-6 (15.6)
35	C20:5n-3	Eicosapentaenoic (EPA)	cis-5,8,11,14,17-Eicosapentaenoic	39.03	Highest peak	C24:1n-9 (60.6)
36	C24:1n-9	Nervonic	cis-15-Tetracosenoic	40.04	Highest peak	C20:5n-3 (60.6)
37	C22:6n-3	Docosahexaenoic (DHA)	cis-4,7,10,13,16,19-Docosahexaenoic	41.68	Highest peak	C24:1n-9 (98.4)

Expected retention time (RT), identification window and identification rule are those of the Xcalibur processing method (TIC trace; ICIS, minimum S/N 3; RT reference). Highest peak, the highest peak within the window is assigned; Nearest RT, the peak nearest to the expected RT is assigned. .

Table S1 (continued). B. Calibration and detection.

No.	Component	Calibration level used for quantification, μg/mL	Linearity range (dilution series), μg/mL	R^2 (dilution series)	LOD, μg/mL	LOQ, μg/mL	Detected, % of 177
1	C4:0	404	10.1–404	0.9929	0.14	0.42	97.7
2	C6:0	404	10.1–404	0.9761	0.29	0.90	60.5
3	C8:0	404	10.1–404	0.9968	0.10	0.38	60.5
4	C10:0	408	10.2–408	0.9943	0.12	0.37	49.2
5	C11:0	204	5.1–204	0.9972	0.18	0.36	0.0
6	C12:0	404	10.1–404	0.9715	0.28	0.85	74.0
7	C13:0	203	5–203	0.9822	0.22	0.67	0.0
8	C14:0	404	10.1–404	0.9844	0.20	0.62	86.4
9	C14:1	204	5.1–204	0.9795	0.23	0.72	2.3
10	C15:0	203	5–203	0.9855	0.20	0.61	48.0
11	C15:1	204	5–203	0.9826	0.22	0.66	0.0
12	C16:0	612	15.3–612	0.9604	0.37	1.13	96.6
13	C16:1	204	5.1–204	0.9754	0.26	0.79	87.6
14	C17:0	210	5.2–210	0.9834	0.21	0.65	72.9
15	C17:1	204	5.1–204	0.9771	0.25	0.76	3.4
16	C18:0	408	10.2–408	0.9805	0.23	0.70	55.9
17	C18:1n-9 trans	202	5–202	0.9693	0.29	0.89	93.8
18	C18:1n-9	404	10.1–404	0.9804	0.23	0.70	98.9
19	C18:2n-6 trans	202	5–202	0.9888	0.17	0.53	1.1
20	C18:2n-6	202	5–202	0.9718	0.28	0.85	100.0
21	C20:0	408	10.2–408	0.9809	0.21	0.69	31.6
22	C18:3n-6	203	5–203	0.9745	0.27	0.80	12.4
23	C18:3n-3	204	5.1–204	0.9769	0.25	0.77	15.8

No.	Component	Calibration level used for quantification, µg/mL	Linearity range (dilution series), µg/mL	R^2 (dilution series)	LOD, µg/mL	LOQ, µg/mL	Detected, % of 177
24	C20:1n-9	202	5–202	0.9618	0.38	1.15	0.0
25	C21:0	203	5–203	0.9583	0.34	1.03	0.6
26	C20:2n-6	204	5.1–204	0.9774	0.25	0.76	0.0
27	C22:0	405	10.1–405	0.9797	0.23	0.71	3.4
28	C20:3n-6	204	5.1–204	0.9630	0.32	0.97	90.4
29	C22:1n-9	204	5.1–204	0.9649	0.31	0.95	0.0
30	C20:3n-3	204	5.1–204	0.9846	0.21	0.62	2.8
31	C23:0	203	5–203	0.9639	0.32	0.96	33.3
32	C20:4n-6	202	5–202	0.9636	0.32	0.97	23.2
33	C22:2n-6	204	5.1–204	0.9657	0.31	0.94	2.3
34	C24:0	404	5.1–204	0.9924	0.14	0.43	67.2
35	C20:5n-3	204	5.1–204	0.9920	0.15	0.45	1.1
36	C24:1n-9	204	5.1–204	0.9638	0.32	0.96	1.1
37	C22:6n-3	203	5–203	0.9845	0.21	0.62	7.3

Sample values were quantified against the single calibration level shown (certified reference mixture CRM47885 at its nominal concentration), with the regression forced through the origin. The dilution series was used only to assess linearity and to estimate the limits of detection (LOD = 3.3σ/S) and quantification (LOQ = 10σ/S), where σ is the standard deviation of the response and S the slope; the limits lie below the lowest calibration level and are extrapolated estimates. They characterise the method on the calibration standards and were not applied to the sample values. Units are µg/mL (given as ng/mL in the previous version in error). Detected, proportion of the 177 samples in which a peak with S/N ≥ 3 was found within the window. Included, detected in at least 50% of samples and entered into the composition and statistical analysis.

Table S1b. Intra-day repeatability of serum fatty acid determination in the pooled serum quality-control sample.

Fatty acid	n	Mean, % of detected profile	SD	CV, %
C14:0 (myristic)	5	0.7207	0.0381	5.29
C16:0 (palmitic)	5	32.0536	0.3814	1.19
C16:1 (palmitoleic)	5	0.9534	0.0340	3.57
C17:0 (heptadecanoic)	5	0.2404	0.0143	5.95
C18:0 (stearic)	5	7.1899	0.2329	3.24
C18:1n-9 (oleic)	5	16.3978	0.9084	5.54
C18:2n-6 (linoleic)	5	27.3132	0.8112	2.97

Five aliquots of a pooled serum QC sample, prepared independently, were analysed in five consecutive injections at the start of the analytical sequence on the same day. Repeatability is reported for the seven components present at proportions sufficient for a meaningful coefficient of variation; it was not available for C4:0, C6:0, C8:0, C12:0, C24:0 and C20:3n-6, and the results of the QC injections interspersed through the sequence could not be retrieved. In these injections the C18 region was assigned correctly. SD, standard deviation; CV, coefficient of variation.

Table S2. Detection frequency and group comparisons for all 37 panel components (n = 177; 45 / 114 / 18), in order of elution.

Component	Detected, %	Detected by group, % (C/P/D)	Control	Prediabetes	T2DM	H	p	q
C4:0	97.7	100/96/100	0.059 [0.050–0.074]	0.056 [0.045–0.078]	0.045 [0.035–0.061]	4.14	0.126	0.391
C6:0	60.5	69/57/61	0.076 [0.000–0.296]	0.005 [0.000–0.121]	0.021 [0.000–0.239]	4.29	0.117	0.391
C8:0	60.5	71/57/56	0.008 [0.000–0.012]	0.002 [0.000–0.008]	0.001 [0.000–0.005]	7.13	0.0284	0.180
C10:0	49.2	62/45/44	0.011 [0.000–0.023]	0.000 [0.000–0.012]	0.000 [0.000–0.011]	6.81	0.0333	0.180
C11:0	0.0	0/0/0	0.000 [0.000–0.000]	0.000 [0.000–0.000]	0.000 [0.000–0.000]	—	—	—
C12:0	74.0	84/70/72	0.010 [0.003–0.015]	0.006 [0.000–0.012]	0.005 [0.000–0.009]	6.72	0.0348	0.180
C13:0	0.0	0/0/0	0.000 [0.000–0.000]	0.000 [0.000–0.000]	0.000 [0.000–0.000]	—	—	—
C14:0	86.4	96/82/89	0.726 [0.496–0.984]	0.742 [0.229–0.985]	0.562 [0.384–1.015]	0.98	0.611	0.839
C14:1	2.3	2/3/0	0.000 [0.000–0.000]	0.000 [0.000–0.000]	0.000 [0.000–0.000]	0.48	0.785	0.856
C15:0	48.0	56/47/33	0.035 [0.000–0.103]	0.000 [0.000–0.084]	0.000 [0.000–0.049]	3.16	0.206	0.531
C15:1	0.0	0/0/0	0.000 [0.000–0.000]	0.000 [0.000–0.000]	0.000 [0.000–0.000]	—	—	—
C16:0	96.6	96/96/100	30.017 [26.725–33.458]	29.675 [26.999–32.537]	32.201 [30.560–35.189]	7.00	0.0302	0.180
C16:1	87.6	96/84/89	0.748 [0.408–1.330]	0.938 [0.353–1.522]	0.948 [0.159–1.693]	0.47	0.789	0.856
C17:0	72.9	89/70/50	0.229 [0.044–0.308]	0.163 [0.000–0.286]	0.011 [0.000–0.169]	7.33	0.0256	0.180
C17:1	3.4	2/4/0	0.000 [0.000–0.000]	0.000 [0.000–0.000]	0.000 [0.000–0.000]	1.20	0.55	0.839
C18:0	55.9	47/61/50	0.000 [0.000–12.985]	11.465 [0.000–14.071]	6.368 [0.000–16.560]	1.76	0.415	0.804
C18:1n-9 trans	93.8	93/95/89	0.558 [0.296–8.886]	0.921 [0.221–10.694]	0.615 [0.305–8.760]	0.44	0.801	0.856
C18:1n-9	98.9	100/98/100	13.811 [11.725–15.175]	13.774 [11.586–16.144]	13.958 [10.368–17.218]	0.18	0.913	0.944
C18:2n-6 trans	1.1	2/1/0	0.000 [0.000–0.000]	0.000 [0.000–0.000]	0.000 [0.000–0.000]	0.76	0.684	0.848
C18:2n-6	100.0	100/100/100	30.985 [26.560–34.823]	28.335 [24.553–32.959]	28.373 [23.693–30.305]	4.94	0.0845	0.328
C20:0	31.6	38/29/33	0.000 [0.000–0.049]	0.000 [0.000–0.028]	0.000 [0.000–0.113]	1.05	0.593	0.839
C18:3n-6	12.4	11/14/6	0.000 [0.000–0.000]	0.000 [0.000–0.000]	0.000 [0.000–0.000]	1.21	0.547	0.839
C18:3n-3	15.8	16/16/17	0.000 [0.000–0.000]	0.000 [0.000–0.000]	0.000 [0.000–0.000]	0.00	0.998	0.998
C20:1n-9	0.0	0/0/0	0.000 [0.000–0.000]	0.000 [0.000–0.000]	0.000 [0.000–0.000]	—	—	—
C21:0	0.6	2/0/0	0.000 [0.000–0.000]	0.000 [0.000–0.000]	0.000 [0.000–0.000]	2.93	0.231	0.550
C20:2n-6	0.0	0/0/0	0.000 [0.000–0.000]	0.000 [0.000–0.000]	0.000 [0.000–0.000]	—	—	—
C22:0	3.4	7/3/0	0.000 [0.000–0.000]	0.000 [0.000–0.000]	0.000 [0.000–0.000]	2.28	0.32	0.695
C20:3n-6	90.4	98/88/89	2.690 [1.800–4.111]	2.564 [1.297–3.514]	2.575 [0.970–4.162]	2.18	0.336	0.695
C22:1n-9	0.0	0/0/0	0.000 [0.000–0.000]	0.000 [0.000–0.000]	0.000 [0.000–0.000]	—	—	—
C20:3n-3	2.8	4/3/0	0.000 [0.000–0.000]	0.000 [0.000–0.000]	0.000 [0.000–0.000]	0.95	0.622	0.839
C23:0	33.3	49/25/44	0.000 [0.000–0.482]	0.000 [0.000–0.003]	0.000 [0.000–0.351]	7.99	0.0184	0.180
C20:4n-6	23.2	33/20/17	0.000 [0.000–0.004]	0.000 [0.000–0.000]	0.000 [0.000–0.000]	3.35	0.187	0.527

Component	Detected, %	Detected by group, % (C/P/D)	Control	Prediabetes	T2DM	H	p	q
C22:2n-6	2.3	2/3/0	0.000 [0.000–0.000]	0.000 [0.000–0.000]	0.000 [0.000–0.000]	0.49	0.784	0.856
C24:0	67.2	73/66/61	0.141 [0.000–0.258]	0.088 [0.000–0.252]	0.039 [0.000–0.236]	1.47	0.48	0.839
C20:5n-3	1.1	0/2/0	0.000 [0.000–0.000]	0.000 [0.000–0.000]	0.000 [0.000–0.000]	1.11	0.574	0.839
C24:1n-9	1.1	4/0/0	0.000 [0.000–0.000]	0.000 [0.000–0.000]	0.000 [0.000–0.000]	5.90	0.0523	0.232
C22:6n-3	7.3	9/6/11	0.000 [0.000–0.000]	0.000 [0.000–0.000]	0.000 [0.000–0.000]	0.81	0.668	0.848

Values are median [interquartile range] of the values as supplied by the laboratory (calibration-corrected values of all 37 components; median sum 91.9 [89.0–94.7]), so they differ from the percentages of the quantified profile in Table 2 of the main text. Detected, proportion of samples with a peak of S/N ≥ 3 within the identification window. Groups were compared by the Kruskal–Wallis test (H, statistic); q, Benjamini–Hochberg adjusted p across the 31 components for which the test could be computed; —, not computable because the component was not detected in any sample. These comparisons are descriptive: the inferential analysis is confined to the 13 included components (Tables 2 and 3 of the main text). Values for C18:0 are not interpretable (Note S1.5). * C18:1n-9 trans excluded as a peak-assignment artefact. Shorthand notation: Cx:y, x carbon atoms and y double bonds; n-z, position of the first double bond from the methyl end. Common and systematic names of all components are given in Table S1A.

Table S3. Partial Spearman correlations between the 13 fatty acids and glycemic and cardiometabolic parameters, adjusted for age, sex, body mass index and waist circumference, r (q). **A.** All participants with covariates (controls and prediabetes).

Fatty acid	HbA1c (n = 107)	Fasting glucose (n = 107)	Triglycerides (n = 107)	HDL-C (n = 107)	LDL-C (n = 107)
C4:0	+0.075 (0.751)	+0.184 (0.374)	−0.313 (0.007)	−0.108 (0.437)	−0.178 (0.218)
C6:0	−0.083 (0.751)	−0.054 (0.814)	−0.103 (0.418)	+0.161 (0.253)	+0.137 (0.257)
C8:0	−0.171 (0.592)	−0.147 (0.427)	−0.077 (0.509)	+0.081 (0.529)	+0.103 (0.379)
C12:0	−0.158 (0.592)	−0.167 (0.374)	−0.175 (0.155)	+0.189 (0.224)	+0.024 (0.808)
C14:0	−0.145 (0.592)	+0.038 (0.814)	+0.163 (0.174)	+0.007 (0.940)	+0.057 (0.607)
C16:0	−0.036 (0.827)	−0.076 (0.814)	−0.260 (0.022)	−0.014 (0.940)	−0.240 (0.056)
C16:1	−0.045 (0.827)	+0.072 (0.814)	+0.247 (0.027)	+0.135 (0.361)	+0.092 (0.409)
C17:0	−0.100 (0.751)	+0.023 (0.814)	+0.028 (0.776)	+0.087 (0.529)	+0.129 (0.268)
C18:0 †	+0.069 (0.751)	+0.028 (0.814)	−0.274 (0.018)	+0.044 (0.769)	−0.261 (0.043)
C18:1n-9	+0.008 (0.936)	+0.177 (0.374)	+0.612 (<0.001)	−0.242 (0.157)	+0.141 (0.257)
C18:2n-6	+0.029 (0.827)	−0.127 (0.500)	+0.082 (0.509)	+0.119 (0.412)	+0.389 (<0.001)
C20:3n-6	−0.105 (0.751)	−0.056 (0.814)	+0.070 (0.512)	+0.161 (0.253)	+0.147 (0.257)
C24:0	−0.063 (0.751)	−0.041 (0.814)	+0.132 (0.285)	+0.191 (0.224)	+0.156 (0.257)

Table S3 (continued). B. Biochemically plausible profiles with covariates.

Fatty acid	HbA1c (n = 71)	Fasting glucose (n = 71)	Triglycerides (n = 71)	HDL-C (n = 71)	LDL-C (n = 71)
C4:0	+0.144 (0.729)	+0.168 (0.412)	−0.280 (0.039)	−0.097 (0.498)	−0.181 (0.424)
C6:0	−0.013 (0.915)	−0.056 (0.903)	−0.315 (0.026)	+0.165 (0.345)	+0.002 (0.987)
C8:0	−0.155 (0.729)	−0.157 (0.412)	−0.229 (0.089)	+0.099 (0.498)	−0.063 (0.795)
C12:0	−0.109 (0.729)	−0.158 (0.412)	−0.272 (0.041)	+0.151 (0.345)	−0.094 (0.795)
C14:0	−0.182 (0.729)	−0.015 (0.927)	+0.062 (0.717)	+0.037 (0.760)	−0.201 (0.405)
C16:0	−0.081 (0.729)	−0.030 (0.927)	−0.331 (0.026)	+0.079 (0.557)	−0.220 (0.405)
C16:1	−0.076 (0.729)	+0.011 (0.927)	+0.290 (0.037)	+0.127 (0.423)	−0.054 (0.795)
C17:0	−0.097 (0.729)	−0.047 (0.903)	−0.091 (0.652)	+0.150 (0.345)	−0.051 (0.795)
C18:0 †	+0.143 (0.729)	+0.127 (0.474)	+0.078 (0.670)	−0.171 (0.345)	−0.054 (0.795)
C18:1n-9	+0.059 (0.739)	+0.158 (0.412)	+0.735 (<0.001)	−0.357 (0.029)	−0.072 (0.795)
C18:2n-6	−0.013 (0.915)	−0.300 (0.142)	−0.313 (0.026)	+0.307 (0.060)	+0.347 (0.039)
C20:3n-6	−0.070 (0.729)	−0.158 (0.412)	−0.034 (0.777)	+0.238 (0.198)	−0.073 (0.795)
C24:0	−0.115 (0.729)	−0.129 (0.474)	+0.052 (0.722)	+0.204 (0.288)	+0.017 (0.964)

Partial correlation computed as the Spearman correlation between the residuals of the fatty acid proportion and of the clinical parameter after linear regression on age, sex, BMI and waist circumference. q, Benjamini–Hochberg adjusted p within each clinical parameter across the 13 fatty acids; exact p-values are in Table S7. BMI is not shown as an outcome because it is one of the covariates. No participant with T2DM is included. † Not interpretable (Note S1.5). Shorthand notation: Cx:y, x carbon atoms and y double bonds; n-z, position of the first double bond from the methyl end. Common and systematic names of all components are given in Table S1A.

Table S4. Effect of the C18:1 correction. **A.** Spearman correlations before (14 components including C18:1n-9 trans) and after (13 components) the correction.

Clinical parameter	Fatty acid	ρ before	q before	ρ after	q after	n
HbA1c	C4:0	−0.066	0.483	−0.062	0.485	177
HbA1c	C6:0	−0.103	0.339	−0.102	0.293	177
HbA1c	C8:0	−0.183	0.071	−0.183	0.063	177
HbA1c	C12:0	−0.144	0.198	−0.146	0.138	177
HbA1c	C14:0	−0.081	0.393	−0.092	0.293	177
HbA1c	C16:0	+0.105	0.339	+0.174	0.066	177
HbA1c	C16:1	+0.031	0.740	+0.034	0.656	177
HbA1c	C17:0	−0.221	0.045	−0.218	0.033	177
HbA1c	C18:0 †	+0.088	0.379	+0.095	0.293	177
HbA1c	C18:1n-9	+0.019	0.801	+0.041	0.635	177

**Clinical parameter**	**Fatty acid**	**ϱ before**	**q before**	**ϱ after**	**q after**	**n**
HbA1c	C18:2n-6	−0.182	0.071	−0.210	0.033	177
HbA1c	C20:3n-6	−0.117	0.336	−0.111	0.293	177
HbA1c	C24:0	−0.098	0.339	−0.098	0.293	177
Fasting glucose	C4:0	−0.038	0.770	−0.049	0.596	177
Fasting glucose	C6:0	−0.021	0.781	−0.024	0.755	177
Fasting glucose	C8:0	−0.115	0.338	−0.122	0.230	177
Fasting glucose	C12:0	−0.130	0.300	−0.141	0.230	177
Fasting glucose	C14:0	+0.054	0.666	+0.045	0.596	177
Fasting glucose	C16:0	+0.155	0.277	+0.184	0.093	177
Fasting glucose	C16:1	+0.084	0.466	+0.080	0.455	177
Fasting glucose	C17:0	−0.110	0.338	−0.117	0.230	177
Fasting glucose	C18:0 †	−0.129	0.300	−0.121	0.230	177
Fasting glucose	C18:1n-9	+0.186	0.185	+0.209	0.069	177
Fasting glucose	C18:2n-6	−0.030	0.770	−0.076	0.455	177
Fasting glucose	C20:3n-6	−0.104	0.338	−0.116	0.230	177
Fasting glucose	C24:0	−0.066	0.598	−0.068	0.476	177
BMI	C4:0	−0.040	0.721	−0.022	0.822	107
BMI	C6:0	−0.071	0.721	−0.072	0.679	107
BMI	C8:0	−0.142	0.337	−0.146	0.348	107
BMI	C12:0	−0.066	0.721	−0.071	0.679	107
BMI	C14:0	−0.056	0.721	−0.057	0.731	107
BMI	C16:0	+0.035	0.721	+0.080	0.679	107
BMI	C16:1	+0.146	0.337	+0.155	0.348	107
BMI	C17:0	−0.146	0.337	−0.152	0.348	107
BMI	C18:0 †	−0.045	0.721	−0.030	0.818	107
BMI	C18:1n-9	+0.242	0.167	+0.291	0.030	107
BMI	C18:2n-6	−0.093	0.682	−0.091	0.679	107
BMI	C20:3n-6	−0.183	0.337	−0.186	0.348	107
BMI	C24:0	−0.042	0.721	−0.040	0.802	107
Triglycerides	C4:0	−0.289	0.011	−0.287	0.011	109
Triglycerides	C6:0	−0.127	0.329	−0.126	0.278	109
Triglycerides	C8:0	−0.103	0.401	−0.104	0.364	109
Triglycerides	C12:0	−0.167	0.194	−0.169	0.147	109
Triglycerides	C14:0	+0.179	0.176	+0.192	0.120	109
Triglycerides	C16:0	−0.131	0.329	−0.177	0.141	109
Triglycerides	C16:1	+0.301	0.010	+0.319	0.005	109
Triglycerides	C17:0	+0.039	0.773	+0.040	0.800	109
Triglycerides	C18:0 †	−0.242	0.040	−0.236	0.044	109
Triglycerides	C18:1n-9	+0.590	<0.001	+0.647	<0.001	109
Triglycerides	C18:2n-6	+0.035	0.773	+0.000	1.000	109
Triglycerides	C20:3n-6	+0.005	0.958	+0.008	1.000	109
Triglycerides	C24:0	+0.118	0.343	+0.128	0.278	109
HDL-C	C4:0	−0.056	0.720	−0.078	0.621	109
HDL-C	C6:0	+0.159	0.244	+0.160	0.235	109
HDL-C	C8:0	+0.069	0.667	+0.069	0.621	109
HDL-C	C12:0	+0.156	0.244	+0.155	0.235	109
HDL-C	C14:0	+0.014	0.951	+0.010	0.915	109
HDL-C	C16:0	+0.022	0.951	−0.026	0.915	109
HDL-C	C16:1	+0.092	0.596	+0.080	0.621	109
HDL-C	C17:0	+0.076	0.667	+0.076	0.621	109
HDL-C	C18:0 †	−0.002	0.986	−0.013	0.915	109
HDL-C	C18:1n-9	−0.183	0.244	−0.258	0.088	109
HDL-C	C18:2n-6	+0.173	0.244	+0.164	0.235	109

Clinical parameter	Fatty acid	ϱ before	q before	ϱ after	q after	n
HDL-C	C20:3n-6	+0.209	0.244	+0.205	0.210	109
HDL-C	C24:0	+0.167	0.244	+0.164	0.235	109
LDL-C	C4:0	−0.195	0.147	−0.212	0.071	109
LDL-C	C6:0	+0.109	0.364	+0.112	0.321	109
LDL-C	C8:0	+0.073	0.485	+0.073	0.485	109
LDL-C	C12:0	+0.038	0.697	+0.029	0.764	109
LDL-C	C14:0	+0.092	0.436	+0.101	0.348	109
LDL-C	C16:0	−0.128	0.285	−0.223	0.071	109
LDL-C	C16:1	+0.152	0.226	+0.162	0.154	109
LDL-C	C17:0	+0.146	0.226	+0.150	0.174	109
LDL-C	C18:0 †	−0.264	0.039	−0.279	0.021	109
LDL-C	C18:1n-9	+0.250	0.041	+0.215	0.071	109
LDL-C	C18:2n-6	+0.314	0.012	+0.335	0.005	109
LDL-C	C20:3n-6	+0.153	0.226	+0.161	0.154	109
LDL-C	C24:0	+0.174	0.199	+0.179	0.136	109

Before, composition renormalized over the 14 components detected in at least 50% of samples, including C18:1n-9 trans; after, over the 13 components used in the main text. q, Benjamini–Hochberg adjusted p within each clinical parameter across the fatty acids of the respective panel (14 before, 13 after; the q-values therefore also reflect the change in the size of the family). Of the 78 correlations, |ϱ| increased by more than 0.001 in 48, decreased by more than 0.001 in 23 and changed by no more than 0.001 in 7. Three associations changed status at $q < 0.05$: C18:2n-6 × HbA1c (ϱ = −0.182, q = 0.071 before; ϱ = −0.210, q = 0.033 after); C18:1n-9 × BMI (ϱ = 0.242, q = 0.167 before; ϱ = 0.291, q = 0.030 after); C18:1n-9 × LDL-C (ϱ = 0.250, q = 0.041 before; ϱ = 0.215, q = 0.071 after). † Not interpretable.

Table S4 (continued). B. Kruskal–Wallis comparisons between glycemic groups before and after the correction (n = 45 / 114 / 18).

Fatty acid	H before	p before	q before	H after	p after	q after
C4:0	4.26	0.119	0.243	5.01	0.0815	0.177
C6:0	4.32	0.115	0.243	4.31	0.116	0.216
C8:0	7.25	0.0267	0.168	7.36	0.0253	0.100
C12:0	6.64	0.0361	0.168	6.93	0.0313	0.100
C14:0	1.03	0.599	0.762	1.11	0.575	0.680
C16:0	4.22	0.121	0.243	6.51	0.0385	0.100
C16:1	0.42	0.811	0.873	0.51	0.775	0.781
C17:0	7.67	0.0216	0.168	7.64	0.0219	0.100
C18:0 †	1.62	0.445	0.644	1.94	0.379	0.547
C18:1n-9	0.04	0.98	0.980	0.49	0.781	0.781
C18:2n-6	5.81	0.0547	0.192	7.94	0.0189	0.100
C20:3n-6	2.28	0.32	0.560	2.17	0.339	0.547
C24:0	1.55	0.46	0.644	1.57	0.457	0.594
C18:1n-9 trans (excluded)	0.46	0.794	0.873	—	—	—

q, Benjamini–Hochberg adjusted p across the components of the respective panel. † Not interpretable.

Table S5. Availability of clinical variables by glycemic group, n recorded / n in group.

Variable	Control	Prediabetes	T2DM	Total
Fasting plasma glucose	45/45	114/114	18/18	177/177
HbA1c	45/45	114/114	18/18	177/177
Age	24/45	85/114	0/18	109/177
Sex	24/45	85/114	0/18	109/177
Body mass index	24/45	83/114	0/18	107/177
Waist circumference	24/45	83/114	0/18	107/177
Systolic blood pressure	24/45	83/114	0/18	107/177
Diastolic blood pressure	24/45	83/114	0/18	107/177
Total cholesterol	24/45	85/114	0/18	109/177
HDL cholesterol	24/45	85/114	0/18	109/177

Variable	Control	Prediabetes	T2DM	Total
LDL cholesterol	24/45	85/114	0/18	109/177
Triglycerides	24/45	85/114	0/18	109/177
Creatinine	24/45	85/114	0/18	109/177
Smoking status	24/45	83/114	0/18	107/177
Antihypertensive medication	24/45	83/114	0/18	107/177

Variables not recorded in the clinical dataset are shown as missing; the reasons were not documented (Section 4.1 of the main text). HbA1c was recorded to the nearest whole percentage point for 117 of the 177 participants (23/45 / 83/114 / 11/18 in C / P / D), including 105 of the 109 with a complete clinical profile. Median FPG in participants with versus without a complete clinical profile: controls 4.96 versus 3.85 mmol/L; prediabetes 5.28 versus 3.99 mmol/L; T2DM, no participant with a complete profile (median 7.43 mmol/L).

Table S6. Sensitivity analyses for the principal associations. **A.** Processing subsets and plausibility definitions: Spearman ρ (q; n).

Subset	C18:1n-9 × triglycerides	C16:1 × triglycerides	C18:2n-6 × HbA1c	C18:2n-6 × LDL-C	C17:0 × HbA1c
Full sample	+0.647 (<0.001; 109)	+0.319 (0.005; 109)	−0.210 (0.033; 177)	+0.335 (0.005; 109)	−0.218 (0.033; 177)
Plausible profiles (main definition)	+0.742 (<0.001; 72)	+0.332 (0.014; 72)	−0.272 (0.042; 115)	+0.283 (0.206; 72)	−0.161 (0.416; 115)
Affected samples (C18:1 duplication)	+0.685 (<0.001; 41)	+0.333 (0.108; 41)	−0.247 (0.164; 71)	+0.342 (0.125; 41)	−0.324 (0.075; 71)
Affected samples, plausible	+0.649 (0.046; 18)	+0.241 (0.785; 18)	−0.387 (0.494; 29)	+0.620 (0.039; 18)	−0.151 (0.896; 29)
Unaffected samples	+0.625 (<0.001; 60)	+0.336 (0.029; 60)	−0.251 (0.185; 95)	+0.188 (0.944; 60)	−0.113 (0.714; 95)
Unaffected samples, plausible	+0.784 (<0.001; 48)	+0.449 (0.006; 48)	−0.325 (0.051; 77)	+0.114 (0.985; 48)	−0.145 (0.676; 77)
Alternative: stearic ≤ 0.8 × oleic	+0.796 (<0.001; 55)	+0.369 (0.010; 55)	−0.262 (0.201; 85)	+0.284 (0.115; 55)	−0.141 (0.491; 85)
Alternative: stearic ≤ 1.2 × oleic	+0.726 (<0.001; 85)	+0.319 (0.019; 85)	−0.250 (0.050; 132)	+0.341 (0.018; 85)	−0.138 (0.376; 132)
Alternative: main definition and ≥ 8 components detected	+0.765 (<0.001; 67)	+0.442 (0.001; 67)	−0.277 (0.044; 110)	+0.226 (0.856; 67)	−0.130 (0.459; 110)
Alternative: stearic ≤ oleic only	+0.580 (<0.001; 76)	+0.229 (0.076; 76)	−0.267 (0.038; 122)	+0.313 (0.078; 76)	−0.195 (0.204; 122)
Alternative: plausible, affected samples excluded	+0.749 (<0.001; 54)	+0.417 (0.007; 54)	−0.272 (0.146; 86)	+0.223 (0.618; 54)	−0.163 (0.462; 86)

Spearman correlations with q (Benjamini–Hochberg within the clinical parameter across the 13 fatty acids, recomputed in each subset) and n. Affected samples, C18:1 duplication present (n = 71); unaffected samples, trans component reported without duplication (n = 95); plausible, main definition in Note S1.5. Alternative definitions: criteria (1) and (3) with stearic acid not exceeding 0.8 or 1.2 times oleic acid; the main definition with at least 8 of 37 components detected; criterion (2) alone; and the main definition with the affected samples excluded. Triglyceride and LDL-C analyses include controls and prediabetes only.

Table S6 (continued). B. Inclusion threshold and exclusion of C4:0: Spearman ρ (q; n).

Inclusion threshold (panel size)	C18:1n-9 × triglycerides	C16:1 × triglycerides	C18:2n-6 × HbA1c	C18:2n-6 × LDL-C	C17:0 × HbA1c
25% (17 components)	+0.647 (<0.001; 109)	+0.319 (0.006; 109)	−0.211 (0.041; 177)	+0.330 (0.008; 109)	−0.218 (0.041; 177)
33% (16 components)	+0.648 (<0.001; 109)	+0.319 (0.006; 109)	−0.210 (0.041; 177)	+0.333 (0.007; 109)	−0.217 (0.041; 177)
40% (15 components)	+0.646 (<0.001; 109)	+0.318 (0.006; 109)	−0.211 (0.037; 177)	+0.335 (0.005; 109)	−0.218 (0.037; 177)
50% (13 components)	+0.647 (<0.001; 109)	+0.319 (0.005; 109)	−0.210 (0.033; 177)	+0.335 (0.005; 109)	−0.218 (0.033; 177)
60% (12 components)	+0.646 (<0.001; 109)	+0.324 (0.003; 109)	−0.244 (0.013; 177)	+0.270 (0.027; 109)	−0.224 (0.016; 177)
70% (9 components)	+0.648 (<0.001; 109)	+0.323 (0.002; 109)	−0.246 (0.009; 177)	+0.278 (0.016; 109)	−0.225 (0.011; 177)
C4:0 excluded (12 components)	+0.646 (<0.001; 109)	+0.319 (0.004; 109)	−0.210 (0.030; 177)	+0.335 (0.004; 109)	−0.219 (0.030; 177)

The composition was renormalized over the components detected in at least the stated proportion of samples (C18:1n-9 trans always excluded); 50% is the main analysis. q within the clinical parameter across the components of each panel.

Table S6 (continued). C. Data subsets: correlation with HbA1c, ρ (p; n).

Fatty acid	All, data subset as covariate (partial)	Complete clinical profile	FPG and HbA1c only	FPG and HbA1c only, without T2DM
C17:0	−0.240 (p = 0.00129; 177)	−0.168 (p = 0.0814; 109)	−0.352 (p = 0.00322; 68)	−0.123 (p = 0.395; 50)
C18:2n-6	−0.175 (p = 0.0199; 177)	−0.094 (p = 0.33; 109)	−0.359 (p = 0.00267; 68)	−0.350 (p = 0.0126; 50)

Partial correlation computed as in Table S3, with the data-subset indicator as the only covariate. In the complete-profile subset HbA1c is effectively binary (Table S5); in the subset with FPG and HbA1c only, HbA1c was recorded to two decimal places for 49 of the 50 participants without T2DM.

Table S6 (continued). D. Compositional approaches: Spearman ϱ (p; n).

Subset	Association	Percentage of quantified profile	clr, 13 components	clr, subcomposition (detected in ≥85%)	ALR to C16:0
Full sample	C18:1n-9 × Triglycerides	+0.647 ($p = 3.0 \times 10^{-14}$; 109)	+0.117 (p = 0.226; 109)	+0.110 (p = 0.255; 109)	+0.735 ($p = 4.1 \times 10^{-19}$; 105)
Full sample	C16:1 × Triglycerides	+0.319 ($p = 7.3 \times 10^{-4}$; 109)	+0.388 ($p = 3.0 \times 10^{-5}$; 109)	+0.371 ($p = 7.2 \times 10^{-5}$; 109)	+0.455 ($p = 6.7 \times 10^{-6}$; 90)
Full sample	C18:2n-6 × HbA1c	−0.210 (p = 0.00506; 177)	+0.102 (p = 0.178; 177)	+0.003 (p = 0.97; 177)	−0.267 ($p = 4.1 \times 10^{-4}$; 171)
Full sample	C18:2n-6 × LDL-C	+0.335 ($p = 3.7 \times 10^{-4}$; 109)	−0.046 (p = 0.635; 109)	−0.039 (p = 0.685; 109)	+0.356 ($p = 1.8 \times 10^{-4}$; 106)
Full sample	C17:0 × HbA1c	−0.218 (p = 0.00353; 177)	−0.192 (p = 0.0106; 177)	n.a.	−0.026 (p = 0.775; 124)
Plausible profiles	C18:1n-9 × Triglycerides	+0.742 ($p = 9.2 \times 10^{-14}$; 72)	+0.309 (p = 0.00823; 72)	+0.344 (p = 0.00308; 72)	+0.728 ($p = 4.3 \times 10^{-13}$; 72)
Plausible profiles	C16:1 × Triglycerides	+0.332 (p = 0.0044; 72)	+0.400 ($p = 5.0 \times 10^{-4}$; 72)	+0.402 ($p = 4.6 \times 10^{-4}$; 72)	+0.503 ($p = 1.3 \times 10^{-5}$; 68)
Plausible profiles	C18:2n-6 × HbA1c	−0.272 (p = 0.00322; 115)	−0.006 (p = 0.946; 115)	−0.155 (p = 0.0978; 115)	−0.321 ($p = 4.8 \times 10^{-4}$; 115)
Plausible profiles	C18:2n-6 × LDL-C	+0.283 (p = 0.0159; 72)	+0.068 (p = 0.572; 72)	+0.097 (p = 0.417; 72)	+0.326 (p = 0.00518; 72)
Plausible profiles	C17:0 × HbA1c	−0.161 (p = 0.0863; 115)	−0.140 (p = 0.136; 115)	n.a.	−0.084 (p = 0.406; 99)

clr, centred log-ratio after multiplicative replacement of zeros at 65% of the smallest detected value of each component; subcomposition: C4:0, C14:0, C16:0, C16:1, C18:1n-9, C18:2n-6, C20:3n-6 (C17:0 is not part of it, n.a.); ALR, additive log-ratio to palmitic acid, computed where both components were detected. For C17:0 × HbA1c under clr transformation of the 13 components, q = 0.120 (replacement by half the smallest detected value), 0.134 (65% of it) and 0.138 (multiplicative replacement).

Table S6 (continued). E. Heptadecanoic acid (C17:0): detection, censored and logistic models, coherence, bootstrap and power.

Analysis	Subset	Result (n)
Not detected, n/N (%)	Full sample	48/177 (27%)
Detected, % (control / prediabetes / T2DM)	Full sample	89 / 70 / 50
Detected, %	Affected samples	51 (36/71)
Detected, %	Unaffected samples	91 (86/95)
Detected, %	Plausible profiles	86 (99/115)
C17:0/C16:0 among detected, % of QC sample, median [IQR]	Affected samples	76 [30–125] (36)
C17:0/C16:0 among detected, % of QC sample, median [IQR]	Unaffected samples	114 [74–139] (81)
Spearman ϱ with HbA1c among detected samples	Full sample	−0.020 (p = 0.819; 129)
Spearman ϱ with HbA1c among detected samples	Plausible profiles	−0.048 (p = 0.634; 99)
Spearman ϱ of log(C17:0/C16:0) with HbA1c	Full sample	−0.026 (p = 0.775; 124)
Tobit regression of log C17:0, per 1 SD of HbA1c	Full sample	β = −0.437 [−0.761; −0.112], p = 0.00835 (177; 48 censored)
Tobit regression of log C17:0, per 1 SD of HbA1c	Affected samples	β = −0.871 [−1.599; −0.143], p = 0.019 (71; 35 censored)
Tobit regression of log C17:0, per 1 SD of HbA1c	Unaffected samples	β = −0.028 [−0.297; +0.242], p = 0.841 (95; 9 censored)
Tobit regression of log C17:0, per 1 SD of HbA1c	Plausible profiles	β = −0.209 [−0.512; +0.093], p = 0.175 (115; 16 censored)
Tobit regression of log C17:0, per 1 SD of HbA1c	Plausible, unaffected	β = −0.030 [−0.303; +0.243], p = 0.831 (77; 5 censored)

Analysis	Subset	Result (n)
Logistic regression of detection, per 1% HbA1c	Full sample	OR = 0.59 [0.40; 0.88], p = 0.0105 (177)
Logistic regression of detection, per 1% HbA1c	Full sample, adjusted for sample signal	OR = 0.41 [0.25; 0.67], p = 4.5×10^{-4} (177)
Logistic regression of detection, per 1% HbA1c	Affected and unaffected samples, adjusted for subset	OR = 0.62 [0.39; 0.98], p = 0.0406 (166)
Logistic regression of detection, per 1% HbA1c	Plausible profiles	OR = 0.69 [0.44; 1.08], p = 0.101 (115)
Logistic regression of detection, per 1% HbA1c	Plausible profiles, adjusted for sample signal	OR = 0.42 [0.23; 0.77], p = 0.00488 (115)
Logistic regression of detection, per 1% HbA1c	Unaffected samples	OR = 0.81 [0.40; 1.66], p = 0.571 (95)
Spearman ϱ, log(C15:0/C16:0) vs log(C17:0/C16:0), where both detected	Full sample	+0.750 (p = 1.1×10^{-15}; 80)
Spearman ϱ, log(C15:0/C16:0) vs log(C17:0/C16:0), where both detected	Affected samples	+0.882 (p = 1.3×10^{-6}; 18)
Spearman ϱ, log(C15:0/C16:0) vs log(C17:0/C16:0), where both detected	Unaffected samples	+0.718 (p = 5.0×10^{-11}; 62)
Spearman ϱ, log(C15:0/C16:0) vs log(C17:0/C16:0), where both detected	Plausible profiles	+0.753 (p = 4.5×10^{-13}; 65)
Spearman ϱ with HbA1c, bootstrap 95% CI (5000 resamples); power for ∣ϱ∣ = 0.22	Full sample	−0.218 [−0.356; −0.072] (177); power 84%
Spearman ϱ with HbA1c, bootstrap 95% CI (5000 resamples); power for ∣ϱ∣ = 0.22	Affected samples	−0.324 [−0.528; −0.100] (71); power 45%
Spearman ϱ with HbA1c, bootstrap 95% CI (5000 resamples); power for ∣ϱ∣ = 0.22	Unaffected samples	−0.113 [−0.306; +0.092] (95); power 57%
Spearman ϱ with HbA1c, bootstrap 95% CI (5000 resamples); power for ∣ϱ∣ = 0.22	Plausible profiles	−0.161 [−0.330; +0.023] (115); power 66%
Spearman ϱ with HbA1c, bootstrap 95% CI (5000 resamples); power for ∣ϱ∣ = 0.22	Plausible, unaffected	−0.145 [−0.368; +0.083] (77); power 49%

QC ratio, C17:0/C16:0 in the pooled QC sample (Table S1b). Tobit, censored normal regression of log C17:0 on standardized HbA1c, with non-detected values left-censored at the smallest detected value. Logistic models: detection of C17:0 (yes/no) on HbA1c per 1%, with, where stated, the number of the other 12 panel components detected (sample signal) or the processing subset as covariate; the nominal associations in the signal-adjusted models are not separable from the processing problem, because detection failures are concentrated in the affected samples. Bootstrap, percentile 95% CI from 5000 resamples. Power, two-sided $\alpha = 0.05$, Fisher z approximation, for ∣ϱ∣ = 0.22 (the full-sample estimate).

Table S6 (continued). F. Regression of HbA1c with additional covariates (controls and prediabetes; n = 107).

Fatty acid	Adjusted for age, sex, BMI	+ smoking and antihypertensive medication	n
C17:0	β = −0.139, p = 0.152	β = −0.130, p = 0.191	107
C18:2n-6	β = +0.017, p = 0.859	β = +0.026, p = 0.787	107

Standardized β per 1-SD increase in the fatty acid proportion. Smoking entered as any versus none; antihypertensive medication as yes/no. Diet and lipid-lowering medication were not recorded.

Table S6 (continued). G. Processing subset (duplication present, n = 71, versus trans component reported without duplication, n = 95) and participant characteristics.

Characteristic	Test	p
Glycemic group	χ^2	0.356
HbA1c	Mann–Whitney	0.192
FPG	Mann–Whitney	0.575
Triglycerides	Mann–Whitney	0.209
HDL cholesterol	Mann–Whitney	0.195
LDL cholesterol	Mann–Whitney	0.0407

Characteristic	Test	p
BMI	Mann–Whitney	0.658
Age	Mann–Whitney	0.722
Data subset	χ^2	0.585

No correction for multiple testing; with eight comparisons, the nominal difference in LDL cholesterol does not survive correction.

Table S7. Exact values for the correlation analyses. **A.** Unadjusted Spearman correlations, all participants (Table 4 of the main text).

Clinical parameter	Fatty acid	Coefficient	p	q	n
HbA1c	C4:0	−0.062	0.411	0.485	177
HbA1c	C6:0	−0.102	0.177	0.293	177
HbA1c	C8:0	−0.183	0.0146	0.063	177
HbA1c	C12:0	−0.146	0.0529	0.138	177
HbA1c	C14:0	−0.092	0.226	0.293	177
HbA1c	C16:0	+0.174	0.0204	0.066	177
HbA1c	C16:1	+0.034	0.656	0.656	177
HbA1c	C17:0	−0.218	0.00353	0.033	177
HbA1c	C18:0 †	+0.095	0.207	0.293	177
HbA1c	C18:1n-9	+0.041	0.587	0.635	177
HbA1c	C18:2n-6	−0.210	0.00506	0.033	177
HbA1c	C20:3n-6	−0.111	0.14	0.293	177
HbA1c	C24:0	−0.098	0.193	0.293	177
Fasting glucose	C4:0	−0.049	0.513	0.596	177
Fasting glucose	C6:0	−0.024	0.755	0.755	177
Fasting glucose	C8:0	−0.122	0.107	0.230	177
Fasting glucose	C12:0	−0.141	0.0607	0.230	177
Fasting glucose	C14:0	+0.045	0.551	0.596	177
Fasting glucose	C16:0	+0.184	0.0143	0.093	177
Fasting glucose	C16:1	+0.080	0.29	0.455	177
Fasting glucose	C17:0	−0.117	0.119	0.230	177
Fasting glucose	C18:0 †	−0.121	0.108	0.230	177
Fasting glucose	C18:1n-9	+0.209	0.00534	0.069	177
Fasting glucose	C18:2n-6	−0.076	0.315	0.455	177
Fasting glucose	C20:3n-6	−0.116	0.124	0.230	177
Fasting glucose	C24:0	−0.068	0.366	0.476	177
BMI	C4:0	−0.022	0.822	0.822	107
BMI	C6:0	−0.072	0.462	0.679	107
BMI	C8:0	−0.146	0.134	0.348	107
BMI	C12:0	−0.071	0.47	0.679	107
BMI	C14:0	−0.057	0.562	0.731	107
BMI	C16:0	+0.080	0.413	0.679	107
BMI	C16:1	+0.155	0.111	0.348	107
BMI	C17:0	−0.152	0.117	0.348	107
BMI	C18:0 †	−0.030	0.755	0.818	107
BMI	C18:1n-9	+0.291	0.00234	0.030	107
BMI	C18:2n-6	−0.091	0.35	0.679	107
BMI	C20:3n-6	−0.186	0.055	0.348	107
BMI	C24:0	−0.040	0.679	0.802	107
Triglycerides	C4:0	−0.287	0.00249	0.011	109
Triglycerides	C6:0	−0.126	0.192	0.278	109
Triglycerides	C8:0	−0.104	0.28	0.364	109
Triglycerides	C12:0	−0.169	0.0793	0.147	109
Triglycerides	C14:0	+0.192	0.046	0.120	109
Triglycerides	C16:0	−0.177	0.0653	0.141	109
Triglycerides	C16:1	+0.319	7.3×10^{-4}	0.005	109
Triglycerides	C17:0	+0.040	0.677	0.800	109
Triglycerides	C18:0 †	−0.236	0.0135	0.044	109
Triglycerides	C18:1n-9	+0.647	3.0×10^{-14}	<0.001	109
Triglycerides	C18:2n-6	+0.000	1.000	1.000	109
Triglycerides	C20:3n-6	+0.008	0.937	1.000	109

Clinical parameter	Fatty acid	Coefficient	p	q	n
Triglycerides	C24:0	+0.128	0.186	0.278	109
HDL-C	C4:0	−0.078	0.419	0.621	109
HDL-C	C6:0	+0.160	0.0968	0.235	109
HDL-C	C8:0	+0.069	0.478	0.621	109
HDL-C	C12:0	+0.155	0.109	0.235	109
HDL-C	C14:0	+0.010	0.915	0.915	109
HDL-C	C16:0	−0.026	0.792	0.915	109
HDL-C	C16:1	+0.080	0.411	0.621	109
HDL-C	C17:0	+0.076	0.431	0.621	109
HDL-C	C18:0 †	−0.013	0.892	0.915	109
HDL-C	C18:1n-9	−0.258	0.00676	0.088	109
HDL-C	C18:2n-6	+0.164	0.0884	0.235	109
HDL-C	C20:3n-6	+0.205	0.0323	0.210	109
HDL-C	C24:0	+0.164	0.088	0.235	109
LDL-C	C4:0	−0.212	0.0272	0.071	109
LDL-C	C6:0	+0.112	0.247	0.321	109
LDL-C	C8:0	+0.073	0.448	0.485	109
LDL-C	C12:0	+0.029	0.764	0.764	109
LDL-C	C14:0	+0.101	0.294	0.348	109
LDL-C	C16:0	−0.223	0.02	0.071	109
LDL-C	C16:1	+0.162	0.0916	0.154	109
LDL-C	C17:0	+0.150	0.12	0.174	109
LDL-C	C18:0 †	−0.279	0.00328	0.021	109
LDL-C	C18:1n-9	+0.215	0.0247	0.071	109
LDL-C	C18:2n-6	+0.335	3.7×10^{-4}	0.005	109
LDL-C	C20:3n-6	+0.161	0.0946	0.154	109
LDL-C	C24:0	+0.179	0.0628	0.136	109

Table S7 (continued). B. Partial correlations adjusted for age, sex, BMI and waist circumference (Table 5 of the main text; Table S3A).

Clinical parameter	Fatty acid	Coefficient	p	q	n
HbA1c	C4:0	+0.075	0.443	0.751	107
HbA1c	C6:0	−0.083	0.397	0.751	107
HbA1c	C8:0	−0.171	0.0775	0.592	107
HbA1c	C12:0	−0.158	0.104	0.592	107
HbA1c	C14:0	−0.145	0.137	0.592	107
HbA1c	C16:0	−0.036	0.714	0.827	107
HbA1c	C16:1	−0.045	0.645	0.827	107
HbA1c	C17:0	−0.100	0.306	0.751	107
HbA1c	C18:0 †	+0.069	0.483	0.751	107
HbA1c	C18:1n-9	+0.008	0.936	0.936	107
HbA1c	C18:2n-6	+0.029	0.764	0.827	107
HbA1c	C20:3n-6	−0.105	0.284	0.751	107
HbA1c	C24:0	−0.063	0.52	0.751	107
Fasting glucose	C4:0	+0.184	0.0581	0.374	107
Fasting glucose	C6:0	−0.054	0.584	0.814	107
Fasting glucose	C8:0	−0.147	0.131	0.427	107
Fasting glucose	C12:0	−0.167	0.0862	0.374	107
Fasting glucose	C14:0	+0.038	0.698	0.814	107
Fasting glucose	C16:0	−0.076	0.434	0.814	107
Fasting glucose	C16:1	+0.072	0.463	0.814	107
Fasting glucose	C17:0	+0.023	0.814	0.814	107
Fasting glucose	C18:0 †	+0.028	0.778	0.814	107
Fasting glucose	C18:1n-9	+0.177	0.0689	0.374	107

Clinical parameter	Fatty acid	Coefficient	p	q	n
Fasting glucose	C18:2n-6	−0.127	0.192	0.500	107
Fasting glucose	C20:3n-6	−0.056	0.565	0.814	107
Fasting glucose	C24:0	−0.041	0.678	0.814	107
Triglycerides	C4:0	−0.313	0.00103	0.007	107
Triglycerides	C6:0	−0.103	0.289	0.418	107
Triglycerides	C8:0	−0.077	0.431	0.509	107
Triglycerides	C12:0	−0.175	0.0717	0.155	107
Triglycerides	C14:0	+0.163	0.0936	0.174	107
Triglycerides	C16:0	−0.260	0.00691	0.022	107
Triglycerides	C16:1	+0.247	0.0102	0.027	107
Triglycerides	C17:0	+0.028	0.776	0.776	107
Triglycerides	C18:0 †	−0.274	0.00424	0.018	107
Triglycerides	C18:1n-9	+0.612	2.5×10^{-12}	<0.001	107
Triglycerides	C18:2n-6	+0.082	0.4	0.509	107
Triglycerides	C20:3n-6	+0.070	0.473	0.512	107
Triglycerides	C24:0	+0.132	0.176	0.285	107
HDL-C	C4:0	−0.108	0.269	0.437	107
HDL-C	C6:0	+0.161	0.0972	0.253	107
HDL-C	C8:0	+0.081	0.407	0.529	107
HDL-C	C12:0	+0.189	0.0516	0.224	107
HDL-C	C14:0	+0.007	0.94	0.940	107
HDL-C	C16:0	−0.014	0.887	0.940	107
HDL-C	C16:1	+0.135	0.166	0.361	107
HDL-C	C17:0	+0.087	0.373	0.529	107
HDL-C	C18:0 †	+0.044	0.651	0.769	107
HDL-C	C18:1n-9	−0.242	0.0121	0.157	107
HDL-C	C18:2n-6	+0.119	0.222	0.412	107
HDL-C	C20:3n-6	+0.161	0.0975	0.253	107
HDL-C	C24:0	+0.191	0.0492	0.224	107
LDL-C	C4:0	−0.178	0.067	0.218	107
LDL-C	C6:0	+0.137	0.158	0.257	107
LDL-C	C8:0	+0.103	0.292	0.379	107
LDL-C	C12:0	+0.024	0.808	0.808	107
LDL-C	C14:0	+0.057	0.561	0.607	107
LDL-C	C16:0	−0.240	0.0128	0.056	107
LDL-C	C16:1	+0.092	0.346	0.409	107
LDL-C	C17:0	+0.129	0.186	0.268	107
LDL-C	C18:0 †	−0.261	0.00667	0.043	107
LDL-C	C18:1n-9	+0.141	0.146	0.257	107
LDL-C	C18:2n-6	+0.389	3.4×10^{-5}	<0.001	107
LDL-C	C20:3n-6	+0.147	0.13	0.257	107
LDL-C	C24:0	+0.156	0.109	0.257	107

Table S7 (continued). C. Unadjusted Spearman correlations, biochemically plausible profiles.

Clinical parameter	Fatty acid	Coefficient	p	q	n
HbA1c	C4:0	+0.005	0.962	0.962	115
HbA1c	C6:0	−0.038	0.683	0.808	115
HbA1c	C8:0	−0.120	0.201	0.523	115
HbA1c	C12:0	−0.106	0.258	0.558	115
HbA1c	C14:0	−0.022	0.819	0.887	115
HbA1c	C16:0	+0.143	0.128	0.416	115
HbA1c	C16:1	+0.092	0.33	0.558	115
HbA1c	C17:0	−0.161	0.0863	0.416	115
HbA1c	C18:0 †	+0.089	0.344	0.558	115

Clinical parameter	Fatty acid	Coefficient	p	q	n
HbA1c	C18:1n-9	+0.143	0.128	0.416	115
HbA1c	C18:2n-6	−0.272	0.00322	0.042	115
HbA1c	C20:3n-6	−0.049	0.605	0.786	115
HbA1c	C24:0	−0.055	0.563	0.786	115
Fasting glucose	C4:0	−0.049	0.601	0.781	115
Fasting glucose	C6:0	−0.116	0.216	0.312	115
Fasting glucose	C8:0	−0.204	0.0287	0.075	115
Fasting glucose	C12:0	−0.221	0.0179	0.075	115
Fasting glucose	C14:0	+0.037	0.694	0.820	115
Fasting glucose	C16:0	+0.244	0.0087	0.057	115
Fasting glucose	C16:1	+0.015	0.873	0.946	115
Fasting glucose	C17:0	−0.204	0.0287	0.075	115
Fasting glucose	C18:0 †	+0.006	0.952	0.952	115
Fasting glucose	C18:1n-9	+0.167	0.0748	0.162	115
Fasting glucose	C18:2n-6	−0.268	0.00381	0.050	115
Fasting glucose	C20:3n-6	−0.118	0.208	0.312	115
Fasting glucose	C24:0	−0.121	0.198	0.312	115
BMI	C4:0	−0.059	0.627	0.680	71
BMI	C6:0	−0.071	0.556	0.676	71
BMI	C8:0	−0.152	0.207	0.384	71
BMI	C12:0	−0.083	0.493	0.676	71
BMI	C14:0	−0.074	0.539	0.676	71
BMI	C16:0	−0.068	0.572	0.676	71
BMI	C16:1	+0.241	0.0429	0.173	71
BMI	C17:0	−0.190	0.113	0.246	71
BMI	C18:0 †	+0.221	0.0644	0.173	71
BMI	C18:1n-9	+0.265	0.0252	0.173	71
BMI	C18:2n-6	−0.240	0.0436	0.173	71
BMI	C20:3n-6	−0.219	0.0664	0.173	71
BMI	C24:0	−0.004	0.973	0.973	71
Triglycerides	C4:0	−0.270	0.0218	0.041	72
Triglycerides	C6:0	−0.383	8.9×10^{-4}	0.005	72
Triglycerides	C8:0	−0.249	0.0349	0.053	72
Triglycerides	C12:0	−0.296	0.0117	0.030	72
Triglycerides	C14:0	+0.054	0.652	0.652	72
Triglycerides	C16:0	−0.284	0.0157	0.034	72
Triglycerides	C16:1	+0.332	0.0044	0.014	72
Triglycerides	C17:0	−0.087	0.469	0.554	72
Triglycerides	C18:0 †	+0.246	0.0369	0.053	72
Triglycerides	C18:1n-9	+0.742	9.2×10^{-14}	<0.001	72
Triglycerides	C18:2n-6	−0.378	0.00106	0.005	72
Triglycerides	C20:3n-6	−0.113	0.345	0.449	72
Triglycerides	C24:0	+0.054	0.651	0.652	72
HDL-C	C4:0	−0.078	0.514	0.556	72
HDL-C	C6:0	+0.256	0.0302	0.098	72
HDL-C	C8:0	+0.110	0.357	0.422	72
HDL-C	C12:0	+0.150	0.207	0.337	72
HDL-C	C14:0	+0.067	0.578	0.578	72
HDL-C	C16:0	+0.127	0.288	0.398	72
HDL-C	C16:1	+0.122	0.306	0.398	72
HDL-C	C17:0	+0.155	0.193	0.337	72
HDL-C	C18:0 †	−0.297	0.0113	0.049	72
HDL-C	C18:1n-9	−0.337	0.00374	0.049	72

Clinical parameter	Fatty acid	Coefficient	p	q	n
HDL-C	C18:2n-6	+0.312	0.00762	0.049	72
HDL-C	C20:3n-6	+0.242	0.0403	0.105	72
HDL-C	C24:0	+0.194	0.103	0.222	72
LDL-C	C4:0	−0.214	0.071	0.308	72
LDL-C	C6:0	−0.062	0.605	0.918	72
LDL-C	C8:0	−0.072	0.549	0.918	72
LDL-C	C12:0	−0.070	0.559	0.918	72
LDL-C	C14:0	−0.143	0.229	0.745	72
LDL-C	C16:0	−0.225	0.0569	0.308	72
LDL-C	C16:1	+0.015	0.897	0.972	72
LDL-C	C17:0	−0.021	0.863	0.972	72
LDL-C	C18:0 †	−0.077	0.52	0.918	72
LDL-C	C18:1n-9	−0.000	0.998	0.998	72
LDL-C	C18:2n-6	+0.283	0.0159	0.206	72
LDL-C	C20:3n-6	−0.016	0.896	0.972	72
LDL-C	C24:0	+0.057	0.635	0.918	72

Table S7 (continued). D. Partial correlations, biochemically plausible profiles (Table S3B).

Clinical parameter	Fatty acid	Coefficient	p	q	n
HbA1c	C4:0	+0.144	0.231	0.729	71
HbA1c	C6:0	−0.013	0.914	0.915	71
HbA1c	C8:0	−0.155	0.198	0.729	71
HbA1c	C12:0	−0.109	0.367	0.729	71
HbA1c	C14:0	−0.182	0.128	0.729	71
HbA1c	C16:0	−0.081	0.503	0.729	71
HbA1c	C16:1	−0.076	0.53	0.729	71
HbA1c	C17:0	−0.097	0.422	0.729	71
HbA1c	C18:0 †	+0.143	0.235	0.729	71
HbA1c	C18:1n-9	+0.059	0.626	0.739	71
HbA1c	C18:2n-6	−0.013	0.915	0.915	71
HbA1c	C20:3n-6	−0.070	0.56	0.729	71
HbA1c	C24:0	−0.115	0.34	0.729	71
Fasting glucose	C4:0	+0.168	0.16	0.412	71
Fasting glucose	C6:0	−0.056	0.646	0.903	71
Fasting glucose	C8:0	−0.157	0.19	0.412	71
Fasting glucose	C12:0	−0.158	0.188	0.412	71
Fasting glucose	C14:0	−0.015	0.904	0.927	71
Fasting glucose	C16:0	−0.030	0.804	0.927	71
Fasting glucose	C16:1	+0.011	0.927	0.927	71
Fasting glucose	C17:0	−0.047	0.694	0.903	71
Fasting glucose	C18:0 †	+0.127	0.291	0.474	71
Fasting glucose	C18:1n-9	+0.158	0.187	0.412	71
Fasting glucose	C18:2n-6	−0.300	0.0109	0.142	71
Fasting glucose	C20:3n-6	−0.158	0.189	0.412	71
Fasting glucose	C24:0	−0.129	0.286	0.474	71
Triglycerides	C4:0	−0.280	0.0182	0.039	71
Triglycerides	C6:0	−0.315	0.00752	0.026	71
Triglycerides	C8:0	−0.229	0.0549	0.089	71
Triglycerides	C12:0	−0.272	0.0219	0.041	71
Triglycerides	C14:0	+0.062	0.606	0.717	71
Triglycerides	C16:0	−0.331	0.00483	0.026	71
Triglycerides	C16:1	+0.290	0.0143	0.037	71
Triglycerides	C17:0	−0.091	0.451	0.652	71
Triglycerides	C18:0 †	+0.078	0.516	0.670	71

Clinical parameter	Fatty acid	Coefficient	p	q	n
Triglycerides	C18:1n-9	+0.735	3.1×10^{-13}	<0.001	71
Triglycerides	C18:2n-6	−0.313	0.00791	0.026	71
Triglycerides	C20:3n-6	−0.034	0.777	0.777	71
Triglycerides	C24:0	+0.052	0.666	0.722	71
HDL-C	C4:0	−0.097	0.421	0.498	71
HDL-C	C6:0	+0.165	0.169	0.345	71
HDL-C	C8:0	+0.099	0.409	0.498	71
HDL-C	C12:0	+0.151	0.209	0.345	71
HDL-C	C14:0	+0.037	0.76	0.760	71
HDL-C	C16:0	+0.079	0.514	0.557	71
HDL-C	C16:1	+0.127	0.293	0.423	71
HDL-C	C17:0	+0.150	0.212	0.345	71
HDL-C	C18:0 †	−0.171	0.155	0.345	71
HDL-C	C18:1n-9	−0.357	0.00223	0.029	71
HDL-C	C18:2n-6	+0.307	0.00922	0.060	71
HDL-C	C20:3n-6	+0.238	0.0457	0.198	71
HDL-C	C24:0	+0.204	0.0886	0.288	71
LDL-C	C4:0	−0.181	0.13	0.424	71
LDL-C	C6:0	+0.002	0.987	0.987	71
LDL-C	C8:0	−0.063	0.603	0.795	71
LDL-C	C12:0	−0.094	0.437	0.795	71
LDL-C	C14:0	−0.201	0.0934	0.405	71
LDL-C	C16:0	−0.220	0.0648	0.405	71
LDL-C	C16:1	−0.054	0.656	0.795	71
LDL-C	C17:0	−0.051	0.672	0.795	71
LDL-C	C18:0 †	−0.054	0.657	0.795	71
LDL-C	C18:1n-9	−0.072	0.548	0.795	71
LDL-C	C18:2n-6	+0.347	0.00301	0.039	71
LDL-C	C20:3n-6	−0.073	0.544	0.795	71
LDL-C	C24:0	+0.017	0.89	0.964	71

Coefficient, Spearman ϱ (A, C) or partial correlation r (B, D). q, Benjamini–Hochberg adjusted p within each clinical parameter across the 13 fatty acids. HbA1c and FPG include all three glycemic groups; the other parameters include controls and prediabetes only. Correlations reaching $q < 0.05$ only in the plausible profiles (panels C and D) are reported for completeness and are not interpreted in the main text. † Not interpretable (Note S1.5). Shorthand notation: Cx:y, x carbon atoms and y double bonds; n-z, position of the first double bond from the methyl end. Common and systematic names of all components are given in Table S1A.
